# Clinical exome sequencing—Mistakes and caveats

**DOI:** 10.1002/humu.24360

**Published:** 2022-03-15

**Authors:** Jordi Corominas, Sanne P. Smeekens, Marcel R. Nelen, Helger G. Yntema, Erik‐Jan Kamsteeg, Rolph Pfundt, Christian Gilissen

**Affiliations:** ^1^ Department of Human Genetics Radboud University Medical Center Nijmegen the Netherlands; ^2^ Donders Institute for Brain, Cognition and Behaviour Radboud University Medical Center Nijmegen the Netherlands; ^3^ Radboud Institute for Molecular Life Sciences Radboud University Medical Center Nijmegen the Netherlands

**Keywords:** clinical exome, clinical variant interpretation, genetic diagnostics, next generation sequencing, NGS data analysis, whole exome sequencing

## Abstract

Massive parallel sequencing technology has become the predominant technique for genetic diagnostics and research. Many genetic laboratories have wrestled with the challenges of setting up genetic testing workflows based on a completely new technology. The learning curve we went through as a laboratory was accompanied by growing pains while we gained new knowledge and expertise. Here we discuss some important mistakes that have been made in our laboratory through 10 years of clinical exome sequencing but that have given us important new insights on how to adapt our working methods. We provide these examples and the lessons that we learned to help other laboratories avoid to make the same mistakes.

## Graphical abstract

Lessons learned from performing clinical exome sequencing for over 10 years.

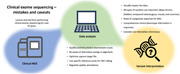

## 1 INTRODUCTION

Massively parallel sequencing technology, or next‐generation sequencing (NGS), has become the standard technique for genetic diagnostics and research. Especially exome and genome sequencing are now applied worldwide to molecularly diagnose patients (Hartman et al., 2019; Marshall et al., 2020; Matthijs et al., 2016). Over the last few years many laboratories have wrestled with the challenges of setting up genetic testing workflows based on a completely new technology. These challenges have been amplified by the fact that sequencing technology has been evolving ever since its introduction with novel instruments, chemistry and analysis methods.

Throughout the past decade, new sequencing technologies have come to market, whereas others have disappeared, and all of them have undergone rapid changes and upgrades (Giani et al., 2020; Heather & Chain, 2016). The same holds true for exome capture kits (Zhou et al., 2021), concomitant equipment and consumables. In this continuously changing field, laboratories have strived to consistently generate high‐quality sequencing data. Various studies have reported how biases in sequencing data may result in either reduced sensitivity or false positive variants for exome and genome sequencing. For example, with NGS, high sequencing error rates and polymerase chain reaction (PCR) duplicates will result in potential false‐positive calls whereas nonuniform sequence coverage or lack of coverage may lead to reduced sensitivity (Barbitoff et al., 2020; Lelieveld et al., 2015). Other issues such as strand bias and insert size distribution may also adversely affect sequencing results (Y. Guo, Li, et al., 2012).

NGS technology is also much more data‐intensive than traditional genetic testing approaches and requires expertise in information technology (IT) and bioinformatics which was initially scarce in many laboratories. Bioinformatics has dealt with the difficulty of setting up rigorous quality control for sequencing data, but also with the challenge of reliable variant identification from sequencing data (Y. Guo et al., 2014). For example, it is relatively difficult to detect insertions and deletions, to identify variants in repeat‐rich or low coverage regions (Jiang et al., 2015; Weißbach et al., 2021), or to distinguish single nucleotide variants (SNVs) from sequencing errors and mapping artifacts. Additionally, the detection of copy number variants (CNVs) from exome data has become a standard procedure giving rise to its own specific challenges (Hong et al., 2016). Similarly, as with sequencing instruments, bioinformatics needs to handle continuous updates from software tools, gene panels and other annotation resources to ensure that molecular geneticists have the latest information available for up‐to‐date data interpretation (Lelieveld et al., 2016). This in turn requires laboratories to implement strategies for automated testing of their analyses as well as systematic approaches for the reanalysis of existing data (Fung et al., 2020; Liu et al., 2019).

Driven by the new sequencing possibilities and the genetic and phenotypic variability of many diseases, clinical genetic testing has changed drastically in the last decade. From targeted gene testing where only one or a few genes would be sequenced based on the clinical phenotype, genetic requests now often concern the analysis of large panels of disease genes. Compared to single gene analysis, the interpretation of the large number of variants from exome or genome sequencing is obviously quite different. This requires not only in‐depth knowledge of the technique to assess the quality of data and identified variants, but also new approaches for variant interpretation. Initial reporting of NGS variants was sometimes too stringent whereby variants that did not exactly match the patient phenotype were omitted, or too lenient, giving rise to many variants of uncertain significance (VUS) (Brownstein et al., 2014; Richards et al., 2015). Over time the quality of the sequencing data has greatly improved and the development of large publicly available databases with variant frequencies, such as the GnomAD database (Karczewski et al., 2020), have helped greatly in the development of more efficient variant filtering options. Moreover, in the last few years various recommendations and quality assessment schemes have been developed that guide the interpretation, classification and reporting of NGS variants (MacArthur et al., 2014; Miller et al., 2021; Rehm et al., 2015; Richards et al., 2015).

There are now several guidelines available on NGS testing, including concrete instructions from the College of American Pathologists (CAP) that can aid in the design, optimization, validation, quality management and bioinformatic aspects of NGS testing (Santani et al., 2019). Nevertheless, many challenges remain and mistakes are bound to happen, even in regulated clinical genetic testing laboratories where quality is of foremost importance. Here we show examples of some of the mistakes that were made in our laboratory throughout 10 years of clinical exome sequencing and the lessons we learned from these mistakes (Table S1). Whereas the wet‐lab has its own particular challenges, here we focus mostly on the issues related to data analysis and variant interpretation. We hope that by sharing these examples other laboratories are safeguarded from making the same mistakes.

## 2 DATA ANALYSIS

Data management and the development of analysis pipelines for sequencing data have become important for many diagnostic laboratories. Building a complete, efficient and robust NGS analysis pipeline is an elaborate task that includes multiple delicate steps from alignment of NGS reads to calling and annotation of different types of genetic variation, such as SNVs, small insertions and deletions, CNVs and short tandem repeats (STRs). Because of the many different processing steps that need to be carried out and the large amount of data, it is relatively easy to make a small mistake with a large but nonobvious impact on the final results. Here we show five examples of mistakes that we made throughout the process of data analysis and that have so far not been abundantly highlighted in literature.

### 2.1 Sequence quality

“Garbage in, garbage out” is a well‐known saying in computer science that captures the concept that flawed input data produces flawed output or “*garbage*.” The same applies to sequencing data. Our laboratory encountered many issues with sequencing results that were not due to mistakes in the processing of the data, but rather due to the fact that there were issues with the initial data generation itself. Identifying the underlying cause of downstream issues can be a challenging task because subtle quality issues in the sequencing data can have large effects on subsequent variant calling. A relatively common issue is data with many spurious variant calls. This happened on occasion due to an unexpected high sequencing error rate, sample contamination, or due to incorrect trimming of adapter sequences (Figure S1). Most of these quality issues can be recognized by inspecting the raw sequencing data or by the observation that called variants have low‐quality scores and deviate from the expected allele fraction of 50% for heterozygous calls. The opposite, a reduced number of variant calls is in most cases due to low sequence coverage. However, there may also be other reasons for reduced sensitivity. In two batches of exome sequencing samples, we noticed a lower number of variant calls only because we performed a trend analysis across several batches of samples. Initially, we expected this to be due to lower sequence coverage of the samples (Figure S2). However, the sequence coverage for these samples was not different from that of other samples. Eventually we discovered that this problem was due to a 10%–20% increase of the fraction of duplicate reads. Because duplicate reads could be due to PCR amplification, and potentially introduce false‐positive variant calls, most variant callers will not consider them for variant calling. Therefore, the effective coverage for many regions was 10%–20% lower than what it appeared in these two batches (Figure S2).

Many quality issues can be readily identified by using tools such as Qualimap that compute quality statistics for sequencing experiments, such as coverage statistics, sequencing error rate, and the percentage of duplicate reads (García‐Alcalde et al., 2012). Therefore, we strongly recommend to embed extensive quality control at all steps of the bioinformatic pipeline and follow trends of quality parameters such as percentage of duplicate reads, coverage distribution, overall number of variants called and percentage of rare variants not found in gnomAD. Deviations from expected values should be investigated closely. Establishing quality thresholds during development and testing will help to identify quality issues later on (Roy et al., 2018; Santani et al., 2019). These thresholds may need to be updated when laboratory protocols are changed, for example with the introduction of new sequencing instruments. A comprehensive quality control analysis on sequencing data can prevent many downstream issues with data interpretation.

### 2.2 Sequence alignment: Alternate contigs

**Figure 1:**
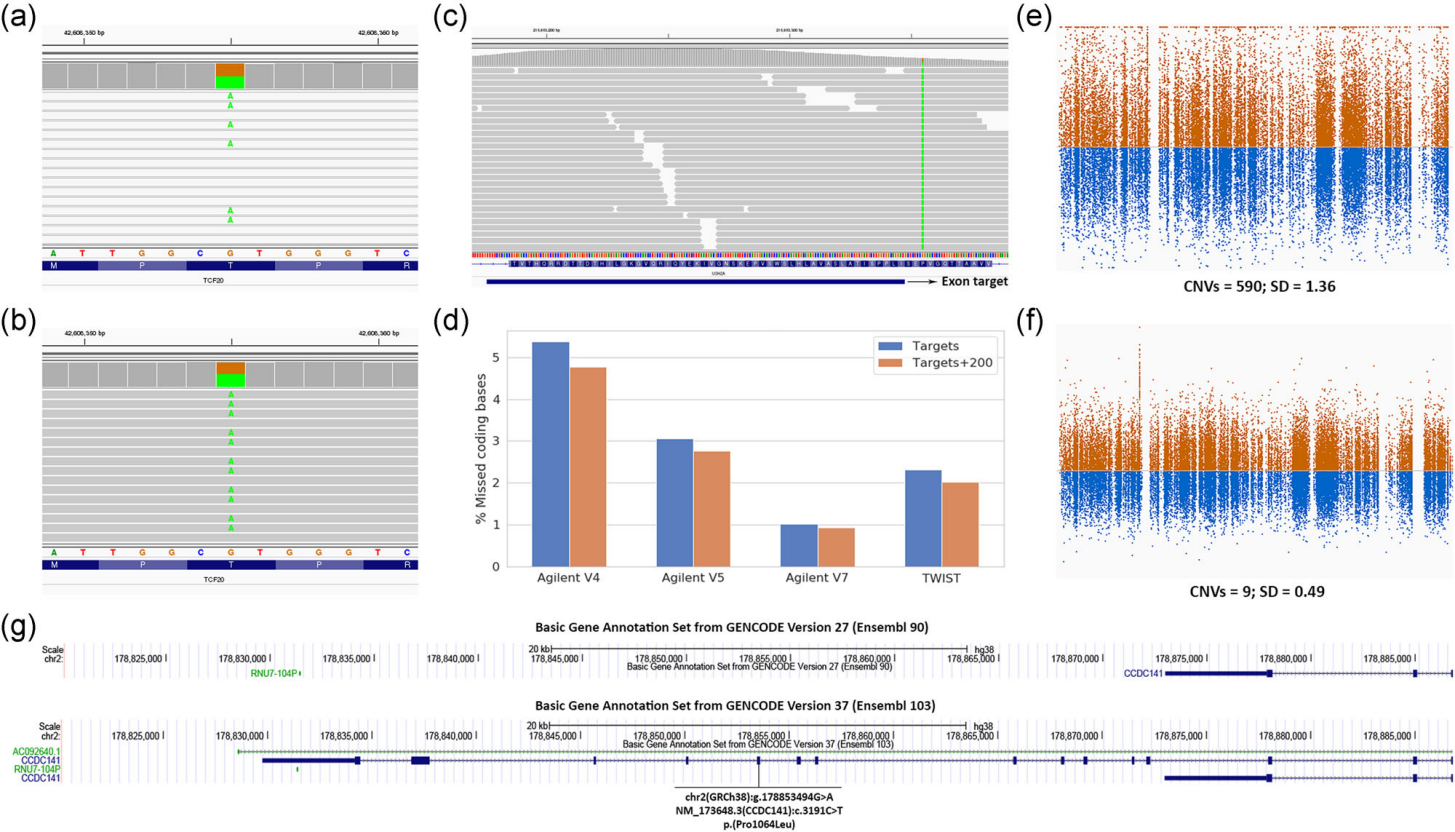
Issues that were encountered in data analysis. (a) IGV screenshot of sequence alignment for a pathogenic coding variant in the gene *TCF20* that was initially not detected because the sequencing reads align to multiple locations in the reference genome due to the inclusion of alternate contigs. (b) Reanalysis of the same sample while excluding alternate contigs led to unique alignments of the sequence reads and detection of this variant. (c) Example of a coding exon where a variant may be missed because the capture target (Agilent SureSelect v5) does not fully overlap with the exonic region. (d) Overview of the percentage of coding bases (Gencode Basic v.34lift37) that is not exactly within the capture targets, and within 200 bp vicinity of the capture targets, for different enrichment methods. (e) Normalized coverage of capture targets (Agilent v5) for an exome sample when using a heterogeneous reference cohort for CNV calling (CoNIFER). Information related to the number of CNVs and autosomal standard deviation (SD) is added to capture the effects of using a heterogeneous reference cohort. (f) The same sample analyzed with a more homogenous reference cohort showing a reduced variation and less CNV calls. (g) UCSC genome browser gene view showing gene structure and transcripts for the gene *CCDC141* for two different GENCODE versions highlighting how additional coding exons may be added that can change variant interpretation. An exome variant is indicated in exon five of the transcript present only in GENCODE version 37. CNV, copy number variants; IGV, Integrated Genomics Viewer

The most primary processing step in NGS data is the alignment of reads to a reference genome. The genome structure of particular regions may, however, vary considerably between different individuals and populations. To properly represent these loci the reference genome makes use of alternate contigs, that is, different reference sequences for particular regions in the genome. These alternate contigs contain regions in the genome that vary in such complex ways that they cannot be represented as a single reference sequence. In our initial analysis workflow we attempted to be as comprehensive as possible and included the largest possible reference genome, that included alternative contigs. However, most read mapping algorithms will, by default, assign a poor mapping quality score to reads that align equally well to multiple regions in the reference genome. These reads with mapping quality (MAPQ) equal to zero are typically indicated in the Integrated Genomics Viewer (IGV; Robinson et al., 2011) with blank reads (Figure 1a). Variant calling algorithms, in turn, will ignore such reads and will not identify variants in regions where reads have low MAPQ scores. Variants in such regions, although visible by manual inspection, will not be called. This mistake was identified with the help of laboratory specialists that looked into the aligned sequencing data to identify whether there was a potential second mutation in a recessive gene (see Section 3.3). We found that by including alternative contigs the number of coding bases where reads cannot be unambiguously aligned will triple. The same issue was recently reported for data from the UK Biobank where the introduction of an important number of alternate contigs in GRCh38 reference genome caused the absence of thousands of variants (Jia et al., 2020). There are two ways in which this problem can be circumvented. The straightforward solution is to simply exclude alternative‐contigs in the analysis, which is currently what is done in our own analysis for exomes on GRCh37. Analyzing the data without alternate contigs will properly align reads in the primary assembly of the human reference genome (Figure 1b). A more sophisticated solution is to apply alignment algorithms that can handle alternate contigs using the corresponding index file which we now do for genomes analyzed using the GRCh38 build of the reference genome (Jia et al., 2020). Considering that GRCh38 greatly expands the repertoire of alternative contigs (among other improvements), it would be advantageous for the clinical community to start transitioning towards GRCh38 to be able to properly detect and analyze genomic variation in population‐specific haplotypes.

### 2.3 Variant calling: Capture target file

There are many different exome kits available that all use their own definition of “regions of interest” (Lelieveld et al., 2015; Pengelly et al., 2020). The naive approach for calling variants from an exome would be to call genome‐wide, without considering capture targets or coding regions. However, this is computationally burdensome, and the resulting data will contain many low‐quality variant calls from off‐target reads in regions that are not of interest. Therefore, it seems reasonable to restrict the analysis to regions where sufficient coverage for reliable variant calling can reasonably be expected. Although the original exome kits tried to exactly target the coding regions, many manufacturers started to move capture probes such that they would be partly overlapping or close to the exon of interest, to optimize the enrichment efficiency. The idea behind this is that a combination of the length of the sequence reads (typically 100–150 bp) and the enrichment of genomic DNA fragments extending beyond but overlapping the targets, will result in sufficient coverage not just for the capture target itself but also for the 100–150 adjacent bases. This indeed improves the capture efficiency for many “difficult” exons but makes it more difficult to decide in which regions variants should be called.

With our initial implementation of a new exome capture design we made the mistake of calling variants only in the exome capture targets, not realizing that a proportion of exons was not directly covered by any capture target, and thereby missing relevant coding variants (Figure 1c). Although we performed several quality checks when testing the exome kit, we did not immediately realize that we were missing as much as 5.4% (1897 kb) of all coding bases (Agilent SureSelect version 4). Again, this mistake was observed when variants that were visible in the sequence alignment through IGV were not present in the variant call files. In more recent exome kits the number of coding bases adjacent to a capture target is less but still considerable (Figure 1d).

Most manufacturers and studies guarantee sufficient coverage 100 bp adjacent to a capture target (Pengelly et al., 2020), but we currently extend our targets with 200 bp, balancing the additional compute time and additional variants called in coding regions. Obviously calling variants genome‐wide will circumvent these issues, but we have judged that the additional compute time and increase in low‐quality variant calls does not make this sufficiently worthwhile. We estimated that calling variants genome‐wide would double analysis time and would yield many more variants called, of which an important number would be artifacts.

When implementing a new exome capture design it is highly recommended to define the clinical targets or regions of interest beforehand and then determine completeness of coverage for these intervals (Matthijs et al., 2016).

### 2.4 Exome CNV calling: Reference pools

Early onwards it became clear that WES can also be used to infer CNVs, based on deviations in sequence coverage between samples (Marchuk et al., 2018). Comparison of coverage between exomes is hampered by coverage biases of individual targets due to sequence capture and GC content (Fromer et al., 2012). Most tools for the detection of CNVs from exome data rely on the creation of a reference pool to standardize the depth of coverage per region and overcome coverage biases in the data (Krumm et al., 2012; Plagnol et al., 2012; Sathirapongsasuti et al., 2011). We discovered that the size and quality of the reference pool has a large impact on the quality of CNV calls. Reference pools with small numbers of samples or a mix of samples with different sequencing characteristics, will lead to increased variability on expected coverage for sequencing targets (Figure 1e). This will result in many spurious calls, making the interpretation much more laborious. In 2016 we accidentally combined samples of which reads were aligned using two different methods in the same reference pool. Unexpectedly this resulted not only in spurious CNV calls but also in large CNVs that were missed but that had already been detected in a previous CNV analysis. Currently, our CNV reference pools are continuously updated using the latest samples, to have minimal technical variability due to changes in sequencing chemistry and protocols (Figure 1f). Besides this continuous updating several separate reference pools are used that match samples based on sequencing platform, enrichment platform, and sex for calling CNVs on chromosome X. To pick‐up on potential quality issues we monitor the number of CNV calls per sample and sequencing batch as well as the average variability of the normalized target coverage per sample in our trend analysis. Based on our experience we would recommend to use a reference cohort for CNV calling that is matched for the capture kit, sequencing instrument and chemistry, and sex.

### 2.5 Annotation: Gene definitions

Whereas we perform regular updates of reference datasets such as population frequencies, OMIM information and HGMD/ClinVar classification, we initially did not regularly update our gene definitions, naively expecting that all genes and transcripts in the human genome have been thoroughly charted. Gene definitions are the most basic resource for the interpretation of genetic variants. Several publicly available resources for gene definition exist, such as RefSeq (developed by the National Center for Biotechnology Information (NCBI)) (Pruitt et al., 2014) and GENCODE that combines a manual annotation by the HAVANA group with computational annotation by Ensembl (Harrow et al., 2012).

When we updated our 2017 GENCODE BASIC gene definitions to a more recent version, somewhat to our surprise we encountered several variants that were initially annotated as noncoding, but that turned out to be in a newly annotated exon, thereby potentially completely changing the interpretation, for example as with the gene *CCDC141* (Figure 1g). There are still regular updates from RefSeq and GENCODE that change known gene definitions and that can have a profound impact on the interpretation of variants for WES. Especially for WGS using more extensive gene definitions can be worthwhile since variants are detected genome‐wide and are not limited to predefined regions as with WES. These ongoing improvements are nicely illustrated by the regular GENCODE updates. GENCODE was updated four times in the last 12 months, and the latest Gencode V38, May 2021 update includes more than 2500 new protein‐coding transcripts, together with several modifications in the list of protein‐coding genes compared to version V33 from January 2020 (Table S2). Regular updates (e.g., every 6 months) for all annotations including gene definitions and periodic re‐annotation of existing samples will likely result in additional diagnoses.

## 3 VARIANT INTERPRETATION

**Figure 2:**
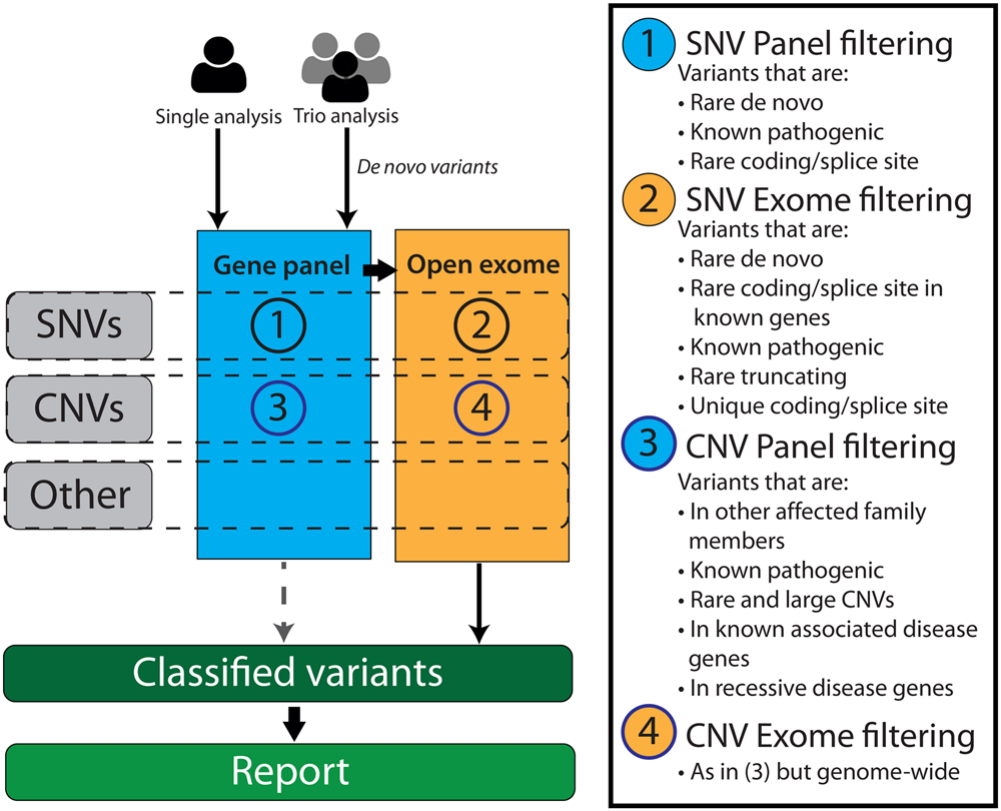
Schematic representation of our interpretation workflow. Gray boxes on the left indicate the analysis of single nucleotide variants (SNVs; GATK calling), copy number variants (CNVs; CoNIFER; Krumm et al., 2012), and ExomeDepth (Plagnol et al., 2012) and “Other,” which includes interpretation of regions of homozygosity (Magi et al., 2014), Uniparental disomy (Yauy et al., 2020), and repeat expansions in coding regions (Dolzhenko et al., 2017)

Next to data analysis, variant interpretation for NGS differs greatly from traditional practices and has come with its own challenges for molecular and clinical geneticists. Here we describe issues that we have encountered and the lessons that we have learned for clinical exome variant interpretation and illustrate these using real‐life examples. These lessons are tentatively in order of importance, starting with what in our experience are the most valuable lessons that we have learned. In all the provided examples, variants were initially interpreted according to our standard protocol which is depicted in Figure 2. We note that in practice these lessons are usually applied in combination, and some examples that we provide could have been used for multiple lessons.

### 3.1 Visually inspect the data

Variant calling algorithms need to balance sensitivity, specificity and performance and will therefore not always provide perfect results (Kumaran et al., 2019). Hence it is good practice to visually inspect sequence alignment data (BAM/CRAM files) to manually filter‐out false positive calls. False‐positive calls often occur in repeat‐rich regions and are readily visible upon inspection of the sequence alignment data. On the other hand, variants and especially insertion/deletion variants may be missed or inaccurately called.

**Figure 3:**
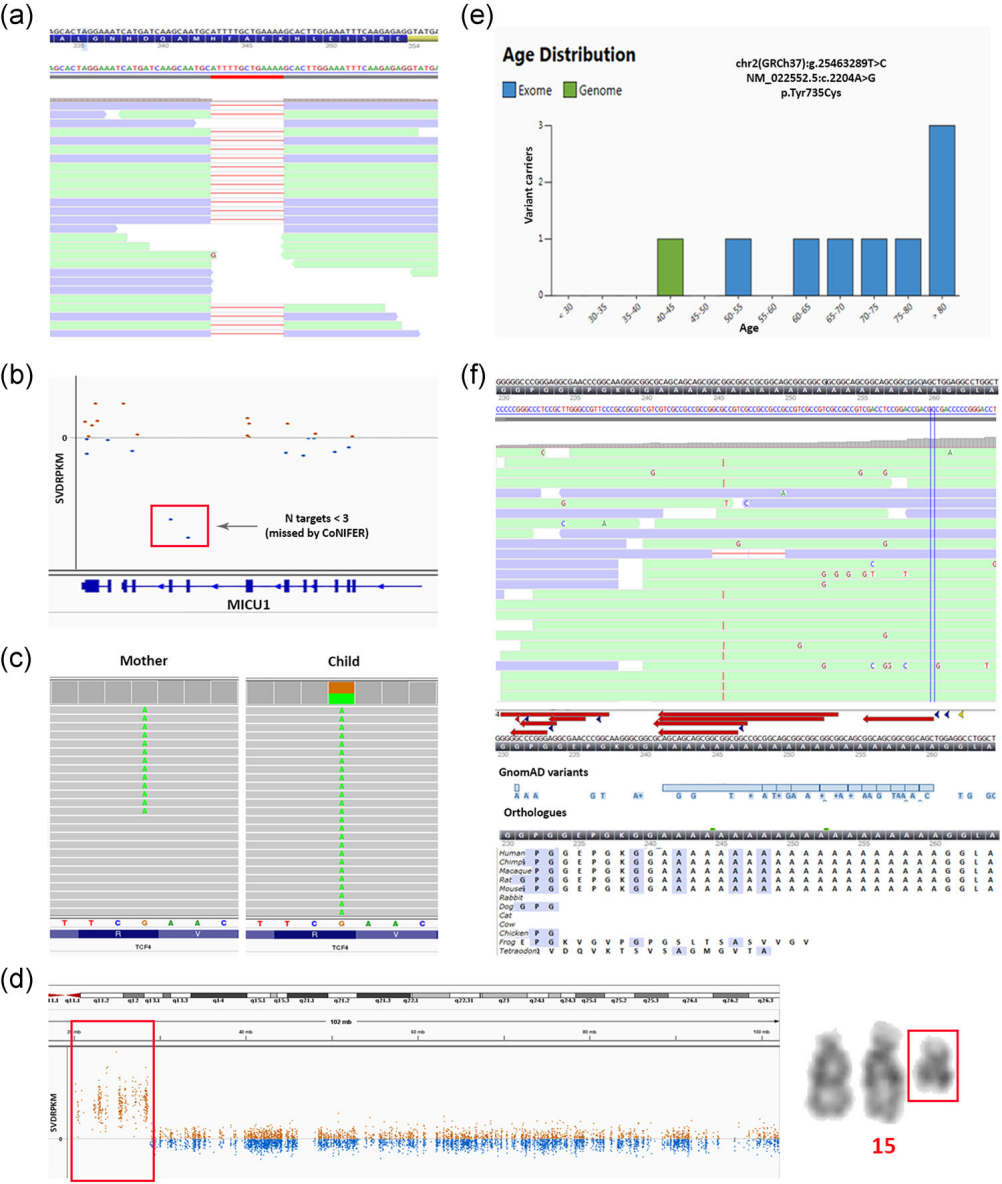
Issues that were encountered in data interpretation. (a) An obvious homozygous deletion in *GPSM2* was incorrectly called by GATK as being heterozygous, indicating that visual inspection of the data is crucial for the correct interpretation of variants. (b) A pathogenic variant in the AR *MICU1* gene was detected together with a two exon deletion in the same gene. (c) A nonsense variant in *TCF4* was not detected when filtering on de novo variants because the mother was a mosaic carrier (30%) of this variant (alignments sorted by base in IGV). (d) An isodicentric marker chromosome (q13.1) was detected in WES data as an ~8.4 Mb terminal gain on 15q11.1q13.1, indicating that it is important to keep the chromosome structure in mind when analyzing WES CNV data. (e) Pathogenic variants in control databases, like the p.(Tyr735Cys) variant in *DNTM3A*, can be recognized by their overrepresentation in older individuals. (f) An 18bp duplication in *PHOX2B* was not called by GATK, but was detected after reanalysis prompted by the distinctive phenotype, emphasizing the power of hypothesis‐driven diagnostics. IGV, Integrated Genomics Viewer

In a patient with a neurodevelopmental disorder we identified two separately called de novo variants (NM_001271.4:c.4592+37del and NM_001271.4:c.4592+38C>G) in the gene *CHD2* (Figure S3). Individually each of these variants is predicted to have a benign or modest effect on splicing, and both variants were initially disregarded. However, after inspection of the alignment data it was clear that this represents a single variant, Chr15(GRCh37):g.93552590_93552591delinsG NM_001271.4:c.4592+37_4592+38delinsG that introduces a new donor splice site predicted to lead to partial intron retention and a premature nonsense variant. Similarly, through visual inspection of the alignment data we found that a 13 base pair heterozygous deletion in *GPSM2* was actually present in a homozygous state (Figure 3a), and was inherited from both parents who were heterozygous for the variant.

Especially in the case of CNVs detected in WES data, visual inspection (of normalized coverage profiles and BAM files) is crucial. For example, a duplication event in the *MTMR2* gene can be recognized as a retrotransposon, that is, the insertion of copy‐DNA in the genome (Huang et al., 2010), by the fact that multiple reads end exactly at the exon–intron boundaries (Figure S4). Similarly, visual inspection is especially important in the case of mosaic deletions, duplications and uniparental disomy, which otherwise could be missed. In a patient with multiple congenital anomalies (left palate, ectopic anus, micropenis, and short proximal limbs) no genetic cause could be discovered with exome sequencing in 2015. However, upon CNV reanalysis of the same data in 2016, we discovered several small copy number gains of which only a few were visible within the restrictions of the requested gene panel. Visual inspection of the normalized coverage profile instantly revealed a gain of the whole short arm of chromosome 12 (Figure S5). The patient was eventually diagnosed with a mosaic quadruplication of the short arm of chromosome 12, causative of Pallister Killian syndrome (OMIM #601803).

Visual inspection of the data is an essential aspect of variant interpretation. There are several tools to do this, including the Integrative Genomics Viewer (IGV) (Robinson et al., 2011). However, visual inspection of data is time‐consuming and should be limited to variants with a higher likelihood of being called incorrectly. Such variants include CNVs, frameshift variants, variants with allele ratios that deviate from the ideal Mendelian ratios (i.e., not clearly heterozygous or homozygous) and multiple adjacent variants in a single gene. In addition, visual inspection should be performed for all variants that the laboratory intends to report.

### 3.2 Variants other than nonsynonymous single nucleotide variants are easily missed

Exome sequencing was originally intended to detect single or multiple nucleotide replacements, or small deletions and duplications (~1–25 bp) within the coding regions and splice sites. In recent years, multiple studies have shown that other types of variants can also, to some degree, be detected in exome sequencing data. This includes among others CNVs (Pfundt et al., 2017), intronic variants (Y. Guo, Long, et al., 2012), uniparental disomies (UPDs) (Yauy et al., 2020), mitochondrial variants (Schlegel et al., 1992), repeat expansions (van der Sanden et al., 2021), and mobile element insertions (Torene et al., 2020). Whereas all of these resolve the cause of disease in only a relatively small number of patients compared to coding single nucleotide variants, together this bycatch can contribute substantially to the diagnostic yield.

For example, routine WES analysis of the coding regions and ±20bp splice site regions did not provide a diagnosis for a leukodystrophy patient with spastic hemiplegia and anarthria. As part of a comprehensive reanalysis by the Solve‐RD consortium (Zurek et al., 2021) a homozygous known pathogenic deep intronic c.1969+115_1969+116del variant in the *CSF1R* gene (Figure S6) was identified that leads to the inclusion of a pseudo‐exon in the *CSF1R* transcripts (L. Guo et al., 2019). Although there was no specific capture target for this region, sequence coverage turned out to be sufficient at this position to call this particular variant.

For a patient with a clinical diagnosis of Stargardt disease, WES analysis of the vision‐disorders panel genes and special focus on the Stargardt genes (*ABCA4* and *ELOVL4*) did not result in a molecular diagnosis. A reanalysis aimed at uniparental disomies detected a paternal isodisomy of chromosome 1 in this patient (Figure S7) (Magi et al., 2014; Yauy et al., 2020). Subsequent Sanger sequencing of the *ABCA4* Stargardt disease gene, located on chromosome 1, uncovered a homozygous pathogenic deep intronic variant (Chr1(GRCh37):g.94546780C>G NM_000350.2(ABCA4):c.859‐506G>C) leading to a pseudo‐exon in a substantial proportion of the *ABCA4* transcripts (Khan et al., 2020).

In a deceased child that suffered from frontal pachygyria, breathing pattern disorders and brachycardia, whole exome analysis was performed. Two rare homozygous variants were detected in the *PLAA* gene, a missense and a synonymous variant. Although initially we focused on the missense mutation it remained a VUS after interpretation. For the synonymous variant, splice prediction tools suggested that it might create an alternative splice donor site in exon 6 of this gene. Since the clinical phenotype of the patient fit with mutations in the *PLAA* gene, subsequent analysis of this predicted splice site effect was requested. Sequencing analysis of complementary DNA that was generated from lymphoblastoid cells from the carrier's parents indeed confirmed the usage of an alternative splice donor site that lead to an out‐of‐frame deletion of 11 nucleotides in the transcript that is encoded by the mutated allele (Figure S8). Instead of “just” being a silent variant, this variant leads to a loss‐of‐function of this allele.

Therefore we would recommend to consider all types of variants within genes that are clinically relevant to the patient's phenotype, and to highlight known pathogenic variants of all types (i.e., independent of their location or frequencies) from databases such as HGMD and ClinVar, during interpretation.

### 3.3 Compound heterozygous variants are easily missed when one of the two is “hiding”

We found that in many cases where recessive inheritance was expected we could initially only identify a single heterozygous (pathogenic) variant in a recessive disease gene, which would be a very good matching gene for the patient's disorder if a second pathogenic variant were to be present. In these cases, the second variant may be a different type of mutation (see Section 3.2), may not meet the quality standards, or may seem less likely to be pathogenic. For example, a heterozygous loss‐of‐function variant, p.(Lys440*), in the *MICU1* gene was detected using standard filtering in a child with a suspicion of a myopathy, based on increased creatine kinase (CK) levels and motor retardation. Only after visual inspection of the CNV data, the second variant, a heterozygous two exon deletion in *MICU1*, was detected (Figure 3b). This CNV was not called by the CNV algorithm (CoNIFER) that was used at the time, since the cut‐off values for calling variants is three or more exons (Krumm et al., 2012).

Another example is the identification of heterozygous loss‐of‐function mutations in the *POLR3A* gene in four unrelated individuals with a movement disorder. Whereas initially these patients received no diagnosis, upon examination we identified an additional intronic variant (NM_007055.4:c.1909+22G>A) in all four patients. The effect of this variant was uncertain, since it is predicted to enhance a cryptic donor splice site, while leaving the original donor splice site intact. This mutation was later shown to be a common hypomorphic variant (i.e., resulting in a milder *POLR3A* phenotype) that results in retention of 19 base pairs in a tissue‐ and stage of development‐specific manner (Minnerop et al., 2017).

These examples demonstrate that when a single heterozygous variant is detected in a recessive disease gene, which could be a good explanation of the patient's phenotype, one should be triggered to take extra efforts to identify a second variant (Kamphans et al., 2013).

### 3.4 Remember mosaicism

Another challenge in the analysis of NGS data that was already alluded to (see Section 3.1) is the occurrence of mosaic SNVs and CNVs. Mosaic SNVs have been shown to be relevant for many disorders. In fact, ~3.5% of variants detected in patients with epilepsy‐related neurodevelopmental disorder were present in a mosaic form (Stosser et al., 2018).

A common practice to remove sequencing and analysis artifacts is to exclude variants with a lower than expected variant allele frequency (VAF). However, such filtering will also remove mosaic SNVs. For example, initial filtering discarded a mosaic (~16%) variant in *PIK3CA* as an artifact in a fetus at 33 weeks of gestation. This pathogenic variant (Chr3(GRCh37):g.178916854G>A NM_006218.4:c.241G>A p.(Glu81Lys)) causes abnormality of the cardiovascular system morphology, which could very well explain the ultrasound abnormalities seen in this fetus. Mosaicism for this variant was confirmed using targeted deep sequencing, revealing ~30% mosaicism allele fraction in the fetus and absence in both parents.

Another challenge arises when a pathogenic variant is also present in a mosaic state in an unaffected parent (Palomares‐Bralo et al., 2017). When performing a trio analysis, the main focus is on the detection of de novo variants in dominant genes. As such, variants that occur in an unaffected (mosaic) parent are not labeled as de novo in the child. Therefore, variants inherited from a mosaic parent, will not be detected when solely looking for de novo variants. For instance, we initially missed a nonsense variant in *TCF4*, Chr18(GRCh37):g.53017619G>A NM_001083962.1:c.520C>T p.(Arg174*), when filtering for *de novo* variants, because 9% of the reads of the mother also contained this variant (Figure 3c). Ideally, such variants would be detected as a separate category when performing a de novo analysis. Alternatively, an inherited variant may be misinterpreted as sporadic due to the low level of mosaicism in a carrier parent, resulting in wrongly estimating the recurrence risk for the parents.

Overall, mosaic variants are not extremely rare. Mosaic variants in genes linked to autosomal dominant‐, autosomal recessive‐, and X‐linked disorders are estimated to occur in 3.3% of individuals whereas parental mosaicism is estimated to be as high as 17.5% of apparently de novo mutations Qin et al. (2016). Whenever considering a potential pathogenic variant relevant to the patient's phenotype, it is worthwhile to also consider the possibility of mosaicism in either the patient or the parents.

### 3.5 Think chromosomes

WES was initially aimed at detecting SNVs and although CNVs can be called from WES data it is important to keep in mind the limitations of WES when interpreting variants. For example, the CoNIFER algorithm does not detect aneuploidies because it normalizes the target coverage per chromosome (Krumm et al., 2012). We initially missed a case of isodisomy X Klinefelter syndrome (XXY) because there were no CNV calls with CoNIFER (the only CNV calling tool used in our lab at that time). Since these were two identical X‐chromosomes, there were Regions of Homozygosity (ROH) calls all over the X‐chromosome, as you would expect in unaffected males. This isodisomy X Klinefelter was discovered using QF‐PCR analysis, but could have been detected sooner by looking at the Y/X coverage ratio in the WES data.

A relatively common copy number finding from WES is the detection of a terminal duplication on one chromosome coinciding with a terminal deletion on another chromosome. This combination is a clear indication of an unbalanced translocation and should be followed up by regular karyotyping. A similar event, a ~265 kb terminal deletion on chromosome 22q13.3, was identified in a patient with severe intellectual disability, developmental delay, absent speech and language, hypotonia and reflux. Since chromosome 22 is an acrocentric chromosome, there were no calls on the short arm of this chromosome. Such a terminal deletion on the long and short arm of the same chromosome is indicative of a ring chromosome. Follow‐up karyotyping revealed that this was indeed a de novo ring chromosome 22 (Figure S9). It is essential to differentiate a ring chromosome from a “regular” terminal aberration since instability during mitosis is a well‐known characteristic of ring chromosomes (Nikitina et al., 2021). Subsequent secondary aberrations, like expansion of the deleted region or even monosomy of the affected chromosome, can have relevant clinical consequences for the affected individual. For chromosome 22 this risk has been described with respect to neurofibromatosis type 2 (NF2; OMIM #607379) where subsequent lifelong routine screening for features of NF2 in these patients is strongly advised (Zirn et al., 2012).

Another example was the identification of a ~8.4 Mb terminal gain on 15q11.1q13.1 in WES data from a patient with intellectual disability and epilepsy. Based on the WES data alone it was not clear whether this gain was caused by an interstitial duplication or by an extra numerical marker chromosome. Upon follow‐up karyotyping this event turned out to be an isodicentric marker chromosome (q13.1) (Figure 3d) and thus in fact was a quadruplication of the q11q13.1 region. This is a clinically relevant finding because tetrasomy 15q gives rise to many nonspecific characteristics including intellectual disability, behavioral disorders, ataxia and epilepsy Finucane et al. (1993).

These examples demonstrate that it is necessary to also have cytogenetics expertize for WES interpretation. Existing guidelines on the interpretation of copy number variants from microarray data can provide guidance for the interpretation and follow‐up of CNVs from exome sequencing data (Shao et al., 2021; Silva et al., 2019).

### 3.6 Genuine disease‐causing variants may be prevalent in population databases

Eliminating common variants is an essential step in exome data filtering (Gilissen et al., 2012). Publicly available databases such as gnomAD that provide aggregated variant information from large populations cohorts are of great help (Karczewski et al., 2020). Commonly used thresholds for such filtering eliminate all data with an allele frequency >1% or based on the frequency and inheritance patterns of the disease (Whiffin et al., 2017). When applying such allele frequency filtering there are a number of reasons why clinically relevant variants may be wrongly discarded.

In a patient with intellectual disability we detected a missense variant in *DNMT3A* (c.2204A>G, p.(Tyr735Cys); NM_022552.5). However, this variant also occurs in 11 individuals in the GnomAD database and therefore was initially considered likely benign. Several studies have now pointed out that particular variants may occur somatically in healthy individuals as a result of clonal hematopoiesis (Acuna‐Hidalgo et al., 2017; Shlush, 2018). Therefore these (somatic) variants occur relatively frequently in control databases where they can be recognized by the fact that they are overrepresented in older individuals (Figure 3e) and have low variant allele fractions Carlston et al. (2017). It is useful to flag such genes that are involved in clonal hematopoiesis. When in doubt, targeted mutation analysis of alternative tissues can help to distinguish between constitutional and somatic variants.

Seemingly frequent pathogenic variants may also be due to homopolymeric stretches. Homopolymeric stretches in genes are regions that are prone to polymerase slippage that can result in the insertion or deletion of a number of nucleotides. These variants may be present in control databases as artifacts, but also may be genuine causative variants in the sequencing data being analyzed. An intriguing example is a deletion or duplication of a single cytosine from a homopolymer stretch of nine nucleotides in the *PRRT2* gene (NM_145239.3:c.641_649) (Figure S10). The subsequent c.649del and c.649dup (rs587778771) variants are present in the gnomAD database with an allele frequency of 0.96% and 0.47%, respectively. These high frequencies initially led us to not consider these variants as a likely cause. However, both events are considered pathogenic, since they lead to frameshifts in the *PRRT2* gene, where haploinsufficiency causes epilepsy, episodic kinesigenic dyskinesia or both. The penetrance of the *PRRT2* related disorders is estimated to be 60% or higher (van Vliet et al., 2012), suggesting that the high allele frequencies of the homopolymer changes in public databases may be due to sequencing artefacts. Indeed, limited alignment data present in gnomAD shows an unequal distribution of the mutant allele in some. It is therefore important to confirm such variants with another test if relevant to the case before reporting.

Although filtering variants using frequency databases is a useful approach, it is not perfect. Again, we would recommend to incorporate safeguards that highlight known pathogenic variants during the data interpretation process to not miss variants with higher populations frequencies (see Section 3.2).

### 3.7 Distinctive clinical features may drive a correct diagnosis

Data analysis may sometimes discard potential variants based on quality criteria. In particular cases, the clinical phenotype can help prioritize variants without the need of additional filtering steps, or can even suggest detailed analysis of specific genes. A de novo 18 bp duplication event in the *PHOX2B* gene was only identified after visual inspection of the sequencing data, which was prompted by the distinctive phenotype of congenital central hypoventilation syndrome in a newborn. This variant was not called, possibly due to poor alignment of sequencing reads in the GC‐rich repetitive sequence of this region (Figure 3f). Interpretation was also a challenge, because the region is not conserved among vertebrates (many lack the repetitive stretch coding for an Alanine repeat) and since many overlapping deletion and duplication events are present in gnomAD. Nevertheless, a duplication event at this position is a recurrent cause of central hypoventilation syndrome.

Another example where a distinct clinical phenotype may help is with identifying highly frequent hypomorphic alleles (see also Section 3.6). We performed a prenatal exome analysis of a fetus with ultrasound anomalies (phocomelia, small chin, prenasal thickness, lower extremities in adducted position) where we at first only detected a paternal 1q21.1 deletion. The fetal phenotype matched with the possible clinical diagnosis of thrombocytopenia‐absent‐radius (TAR) syndrome (Albers et al., 2012). This syndrome is generally caused by a recurrent microdeletion in 1q21.1 in combination with, for example, a hypomorphic variant in the 5′‐untranslated region at position −21 that has an allele frequency of >2% in the gnomAD database. Upon loosening the frequency filtering indeed the variant at position −21 emerged and was of maternal origin.

These examples show how a patient's phenotype may very specifically point to a single gene or a small number of genes. The attention should not only be directed to variants in those genes that may not have been called, but also to other less likely variants, such as silent or deep‐intronic variants that may affect splicing (also see Section 3.2). It is thus beneficial to have dedicated specialists interpreting clinical exome sequencing data of specific groups of disorders, as this allows for deeper knowledge of gene etiologies, atypical variant types, or genotype–phenotype correlations within their area of expertize. The ability to reach a correct diagnosis will however always depend on the availability of complete clinical phenotype information, preferably in a standardized format.

### 3.8 Phenotypic information may be misleading

Whereas phenotypic information is essential for proper genetic testing, it might also hinder the genetic diagnosis by the selection of gene‐targeted tests. With the introduction of NGS techniques such as WES and WGS in genetic labs, the diagnostic strategy of referring clinicians changed from a phenotype‐first to a genotype‐first approach. By more‐or‐less unbiased sequencing analysis it became clear that pathogenic variants in well‐known disease genes can also lead to a very different clinical phenotype depending on the position or type of genetic variation.

Compound heterozygous pathogenic variants in the *IL11RA* gene were detected in a 2‐year‐old child with neonatal hypotonia, feeding problems, myoclonic movements, opsoclonus, frontal bossing, and club feet, and a mitochondrial disorder was suspected. The *IL11RA* gene is, however, involved in “craniosynostosis with dental anomalies” (OMIM #614188). In this rare disorder, no hypotonia or movement disorders were described. Prompted by the finding, a computed tomography scan revealed early closure of the sutures in the child and in a 3‐year‐old sibling. This sibling was then also shown to be compound heterozygous for the *IL11RA* variants. Thus, the frontal bossing, and perhaps the clubfeet, were early indicators of craniosynostosis, while the neurological features may or may not be explained by the *IL11RA* variants.

This kind of phenotypic heterogeneity is of course not new, but NGS implementation has generated many recent examples such as pathogenic *SRCAP* and *CREBBP* variants being causative for Floating Harbor (OMIM #136140) and Rubinstein‐Taybi (OMIM #613684) syndrome, respectively. Variants in these genes have also been described causing a separate syndromic entity depending on the location of the (de novo) loss‐of‐function variant (Menke et al., 2018; Rots et al., 2021). Disease progression, incomplete clinical assessments, or phenotypic heterogeneity may initially be misleading. When detecting obvious pathogenic variants, they should not be set aside as “not compatible with the phenotype” too easily.

### 3.9 Non‐Mendelian inheritance

Most standard filtering strategies for WES data analysis and interpretation are based on classic Mendelian inheritance patterns. Whereas incomplete penetrance is obviously not a new phenomenon in genetic diseases, it does pose a challenge in efficiently filtering large sets of variants from NGS data (Cooper et al., 2013). Especially when handling patient‐(healthy) parent trio data, variant filtering can lead to rejecting inherited heterozygous variants in dominant genes, or rejecting heterozygous X‐linked variants in females of paternal origin or in X‐linked recessive genes.

A trio‐based WES analysis for a young woman with severe intellectual disability, autism and epilepsy initially did not result in a diagnosis. When discussing this result with the referring clinician, the possibility of a variant in the *PCDH19* gene was mentioned. *PCDH19* causes a female‐restricted X‐linked disorder of developmental and epileptic encephalopathy‐9 (OMIM #300088). Targeted inspection of the data indeed revealed a paternally inherited pathogenic variant (ChrX(GRCh37):g.99662889G>A NM_001184880.1:c.707C>T p.Pro236Leu) in the *PCDH19* gene. This missense variant was initially missed because of the inheritance from the healthy hemizygous father. One should thus be aware of heterozygous *PCDH19* variants that may very well be inherited from unaffected hemizygous fathers.

Another challenging group of genes are those that are parentally imprinted, and thus expressed depending on the gender of the parent that passes on the allele. There are approximately 15 well‐described disorders already known to be caused by imprinted loci (Monk et al., 2019), but in addition several hundred genes are known or predicted to be subjected to genomic imprinting (https://www.geneimprint.com/site/home; Monk et al., 2019). In a patient with multiple congenital anomalies we detected a *de novo* frameshift variant in the *IGF2* gene, that is, known to be subjected to imprinting and to be exclusively expressed on the paternal allele. Since genomic phasing information could not be extracted from the WES data of this patient, we were not able to determine on which allele the *IGF2* variant was present. Using an informative SNP (rs368743181) located 3.5 kb upstream of the frameshift variant in combination with genomic phased long‐read sequencing could confirm that this mutation indeed arose on the paternal allele and could therefore be considered as causative. Had this variant not been *de novo*, but inherited from a healthy parent it would have been much more challenging to identify.

Also relevant here is the detection of uniparental disomy events that occur in one in 500–2000 individuals (Nakka et al., 2019; Yauy et al., 2020). In the case of a UPD, both chromosomes are inherited from the same parent and variants in imprinted genes can be a likely cause of disease. Annotation of genes with information about known disease mechanisms can be very useful for interpretation of WES data.

### 3.10 Be aware of isoforms, pseudogenes and gene copies

Our concept of a gene's regulation has long been simplified as a single promoter driving the transcription of a gene, followed by the splicing of the pre‐messenger RNA deleting all introns. Nowadays, we know that gene expression is controlled in a time‐, tissue‐, or developmental stage‐dependent manner. For example, splicing isoforms may lack one or more exons (natural exon skipping), have additional relevant exons (Bodian et al., 2021), have different translation initiation sites, or genes may have multiple promoters causing the occurrence of different isoforms. The difficulty is to consider which isoform is relevant to disease, how to value a variant that is present in just a subset of isoforms, or, in case the reading frame is different between isoforms, how to ensure not missing the relevant “annotation” (Frankish et al., 2015; Schoch et al., 2020).

For example, we identified the Chr19(GRCh37):g.13339572G>A variant in the *CACNA1A* gene in a patient with episodic ataxia. In only one out of five isoforms of *CACNA1A*, this variant is a nonsense variant, NM_001127221.1:c.5569C>T p.(Arg1857*), while it is intronic in the other four (Figure S11). The polyQ expansion track involved in spinocerebellar ataxia type 6 (OMIM #183086) is encoded by two other *CACNA1A* isoforms (NM_001127222.2 and NM_023035.3), suggesting these two isoforms to be essential for proper cerebellar function. Thus, the fact that the nonsense variant is only present in the isoform that does not encode the polyQ track, initially led us to consider this variant as likely benign. However, Graves et al. (2008) showed that this isoform uses an alternative exon 37A instead of the original exon 37B and that nonsense variants in this isoform cause episodic ataxia (OMIM #108500).

Alternatively, isoform‐specific variants may appear pathogenic, but may be benign since the entire isoform is redundant. Finally, some isoforms have partially different reading frames due to exon skipping, making it particularly difficult to annotate variants in them correctly. For variants with different effects in different isoforms all consequences are usually available, but for convenience the most severe consequence is prioritized (e.g., stop‐loss over missense). Nevertheless, this may have consequences for diseases, like Noonan syndrome, with gain‐of‐function or dominant‐negative mechanisms, where missense variants are pathogenic and nonsense variants are not. It is, overall, important to ensure variant calling and annotation in multiple isoforms followed by correct interpretation to not miss the relevant variants.

Also, gene copies and pseudogenes pose a serious problem in WES because of ambiguous sequence alignment of short sequence reads and the subsequent lack of variant calls in such regions. Notorious are copies of complete disease genes, such as *SMN1*, *CYP21A2*, *PKD1, STRC*, or parts of genes, such as the invariant triplicate of eight exons within the *NEB* gene (Donner et al., 2004; Mandelker et al., 2016). However, other variants may be called and display aberrant variant allele fractions, that is, heterozygous when homozygous or very low percentages in heterozygotes, or represent false‐positive calls from the pseudogene(s) as we found for a nonsense variant in the *STRC* gene (Figure S12). One should be made aware of these genes during interpretation based on existing resources and perform validations of the presence and zygosity of such variants if identified, using independent techniques. Different laboratory approaches, such as NGS‐based copy number assessment supplemented with a long‐range PCR‐base Sanger or MiSeq assay (Mandelker et al., 2014), have been suggested for this. In addition, it is possible to simply exclude segmental duplications from the analysis (Santani et al., 2017).

When based on the patient phenotype the detection of known pathogenic mutations could be difficult because of pseudogenes, patients should also be tested in a targeted fashion.

## 4 DISCUSSION

Here we provide some of the most important lessons that we have learned from performing clinical exome sequencing for over 10 years. As a diagnostic laboratory the focus on quality and robustness does not encourage continuous change, but keeping up with updates and innovations has become an essential process in this fast‐evolving field. By providing examples of mistakes that we have made in the development of our diagnostic workflows we hope we can not only create awareness of these specific issues but also of the fact that mistakes *do* occur in diagnostic laboratories. It is essential to be transparent to patients and referring clinicians about the limitations of clinical exome sequencing. These limitations should ideally be mentioned in diagnostic reports Claustres et al. (2014). Although some of the mistakes that were made have required us to recontact patients with a correct diagnosis, we feel that this is partly unavoidable and that a fear of making mistakes should not hamper innovation and improvements as this would do more harm to patient care in the long term.

For this reason, it is however important to have a comprehensive framework for the timely detection of mistakes and problems at the level of the sequencing, data analysis as well as interpretation. Several initiatives can aid laboratories in this by providing benchmark datasets (Zook et al., 2019), and facilitating comparisons between laboratories Muller and European Molecular Genetics Quality Network (2001). An interesting observation from these examples is that issues that occurred during sequencing were sometimes not identified by the sequencing laboratory itself, but rather by the bioinformaticians who analyzed the data. Similarly, mistakes made in data processing were often picked‐up by molecular geneticists during data interpretation. It is therefore essential to have routine procedures for feedback between the members involved in the different parts of the clinical exome sequencing process (i.e., sequencing facility, bioinformatics and data interpretation).

Although it may seem that the examples are very rare exceptions that are unlikely to have much relevance for everyday cases, we would argue that these “exceptions” are alike to rare genetic disorders that may be individually rare, but altogether quite common. It is of course not always feasible to dedicate the amount of time needed to consider all rare possibilities when performing routine exome interpretation. Therefore, data analysis, annotation and procedures should be gradually optimized to increase the automated pickup of such clinically relevant genetic variants. Similarly, it may be a relatively high investment to validate, setup and perform the multitude of possible analyses for WES, such as detection UPDs, mitochondrial variants, repeat expansions, mobile element insertions, and so forth. Data‐sharing and reanalysis efforts such as the Solve‐RD consortium (Zurek et al., 2021) may then prove beneficial and can leverage the large number of samples to perform analyses that are unlikely to diagnose any individual sample but within a large cohort will identify a handful of cases.

The mistakes that we presented here will probably not be our last ones. We strive to learn from our mistakes to improve diagnostics in the long run, and we hope that others can learn from our mistakes as well.

## CONFLICTS OF INTEREST

The authors declare no conflicts of interest.

## Supporting information

Supporting information.

## Data Availability

Data sharing is not applicable to this article as no new data were created or analyzed in this study.
