# Supplementary material for: Clinical exome sequencing—Mistakes and caveats
Source: Hum Mutat. 2022 Mar 15;43(8):1041–55. doi: 10.1002/humu.24360 (PMC9541396; doi:10.1002/humu.24360)
Supplement: Supplementary file 1 — Supporting information. [file HUMU-43-1041-s001.pdf]

Supplementary figures

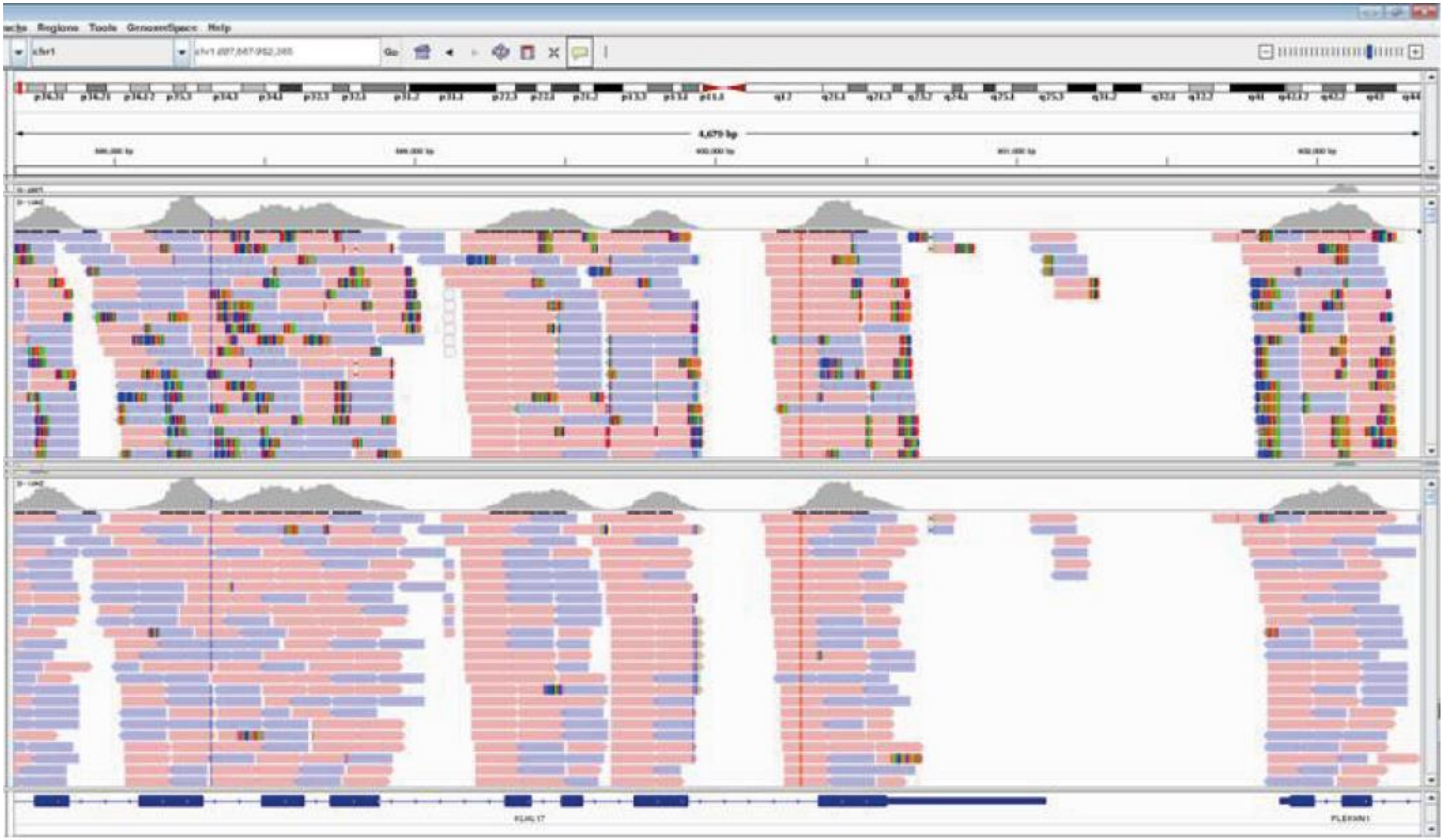

Supp. Figure S1. Poor quality sequencing data due to insufficient adapter trimming. Upper panel shows an IGV screenshot of a sample where adaptors were not trimmed. This can be recognized by a large number of mismatch bases (colored positions in the reads) at the end of reads. Bottom panel shows the same sample after additional trimming.

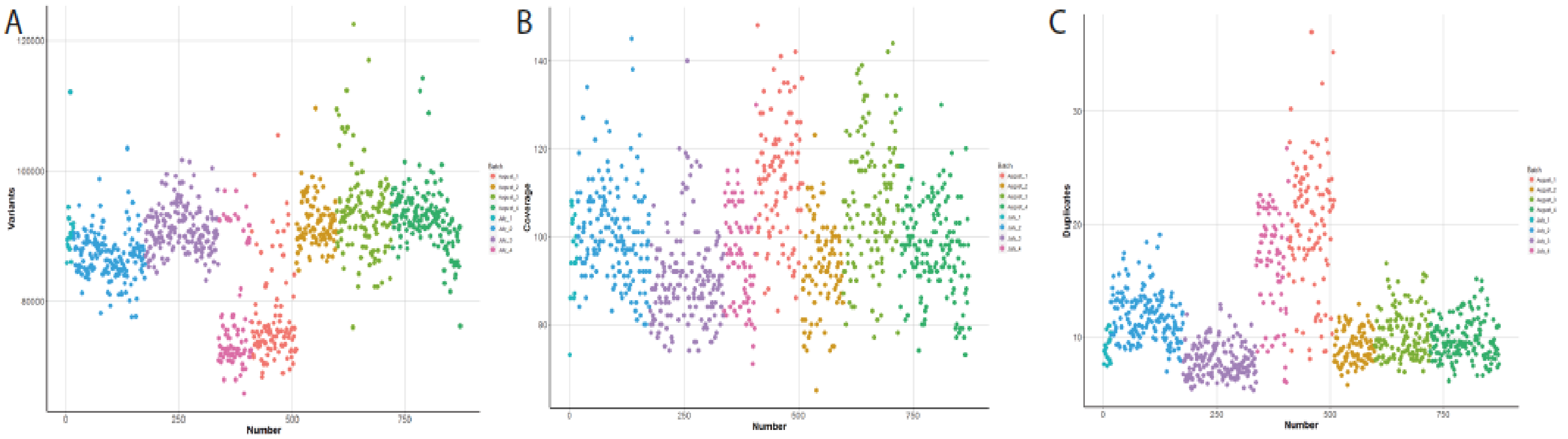

Supp. Figure S2. Missed variants due to a high percentage of duplicate reads. In each image the X-axis shows individual samples sorted by time of sequencing. Each dot indicates one WES sample. Colors indicate different sequencing batches. **A)** Y-axis indicates the number of raw variants called per sample, showing a clear drop in called variants for two batches (purple and red) of samples. **B)** Y-axis indicates the average sample coverage showing that there is not less raw coverage for the purple and red batches of samples. **C)** Y-axis indicates the percentage of duplicate reads per sample. The purple and red samples show a clear increased percentage of duplicate reads which is the cause of the low number of variant calls.

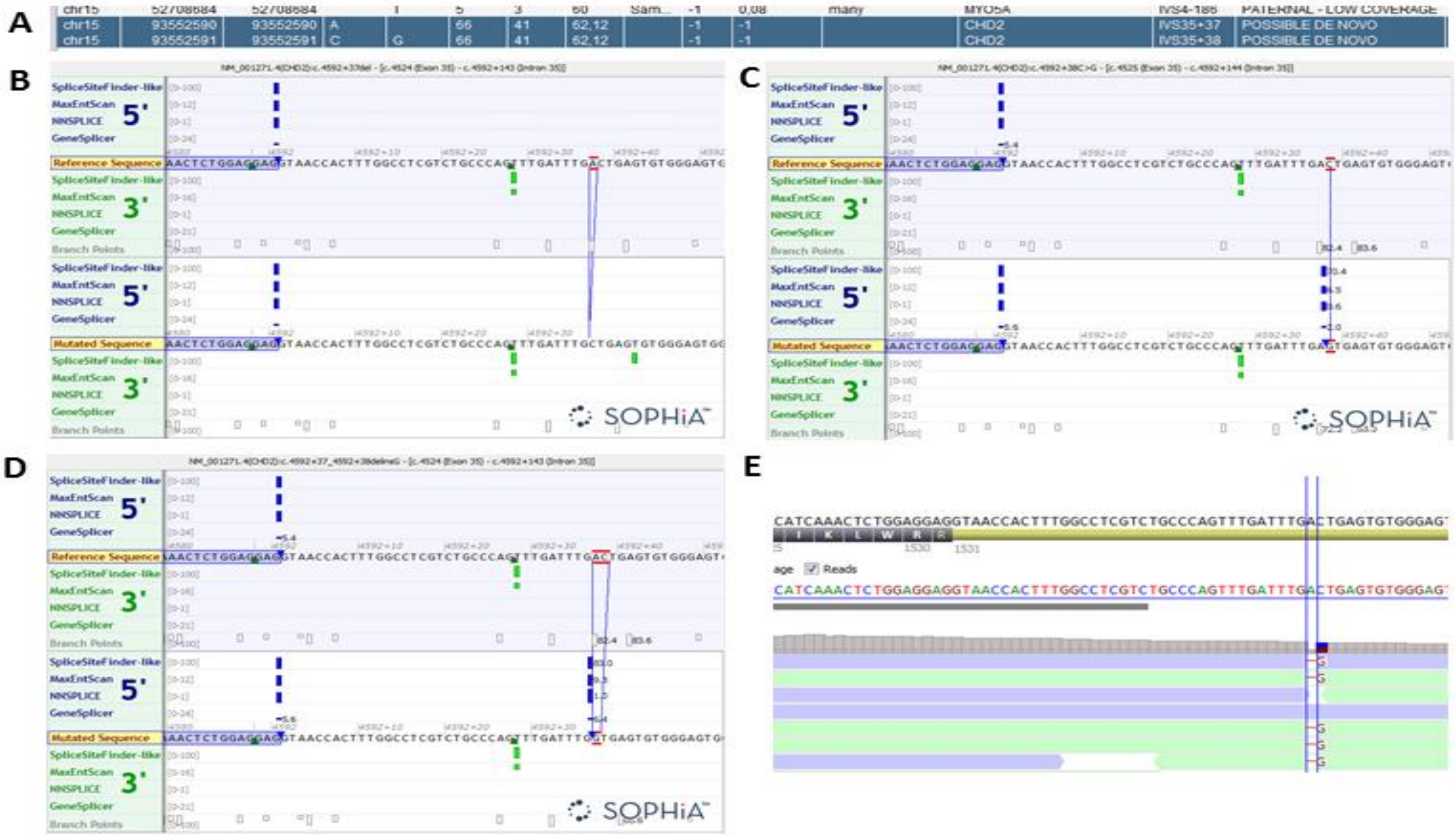

Supp. Figure S3. Alamut interpretation of variants identified in the gene *CHD2*. A) The c.4592+37_4592+38delinsG variant in *CHD2* was indicated in the VCF as two separate variants (c.4592+37del and c.4592+38C>G), **B+C)** which appeared to be harmless or to have a milder effect on the donor splice site on their own. **D)** The pathogenic c.4592+37_4592+38delinsG variant. **E)** can be detected easily by visual inspection of the sequence alignment data (BAM/CRAM file).

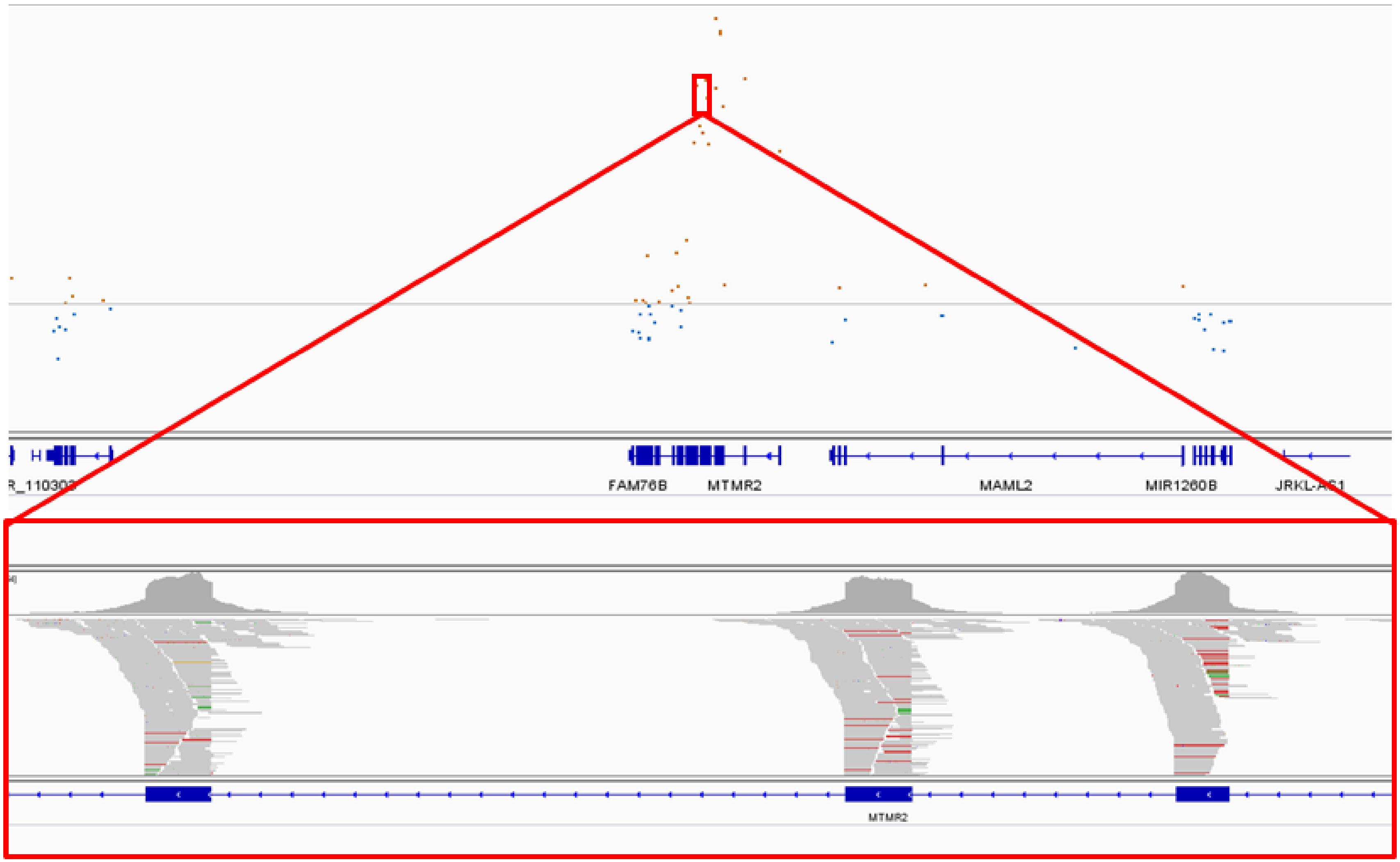

Supp. Figure S4. Retrotransposons of mRNA in the *MTMR2* gene appear as duplication events. Upper panel shows an IGV view of the normalized sequence coverage per exome target (indicated by dots) that seem to indicate a duplication event in the gene MTMR2. Zoom-in (lower panel) shows the alignment of reads on the *MTMR2* exons. Retrotransposons can be recognized as multiple reads that end exactly the exon-intron boundaries, upon visual inspection of the bedgraph and BAM files.

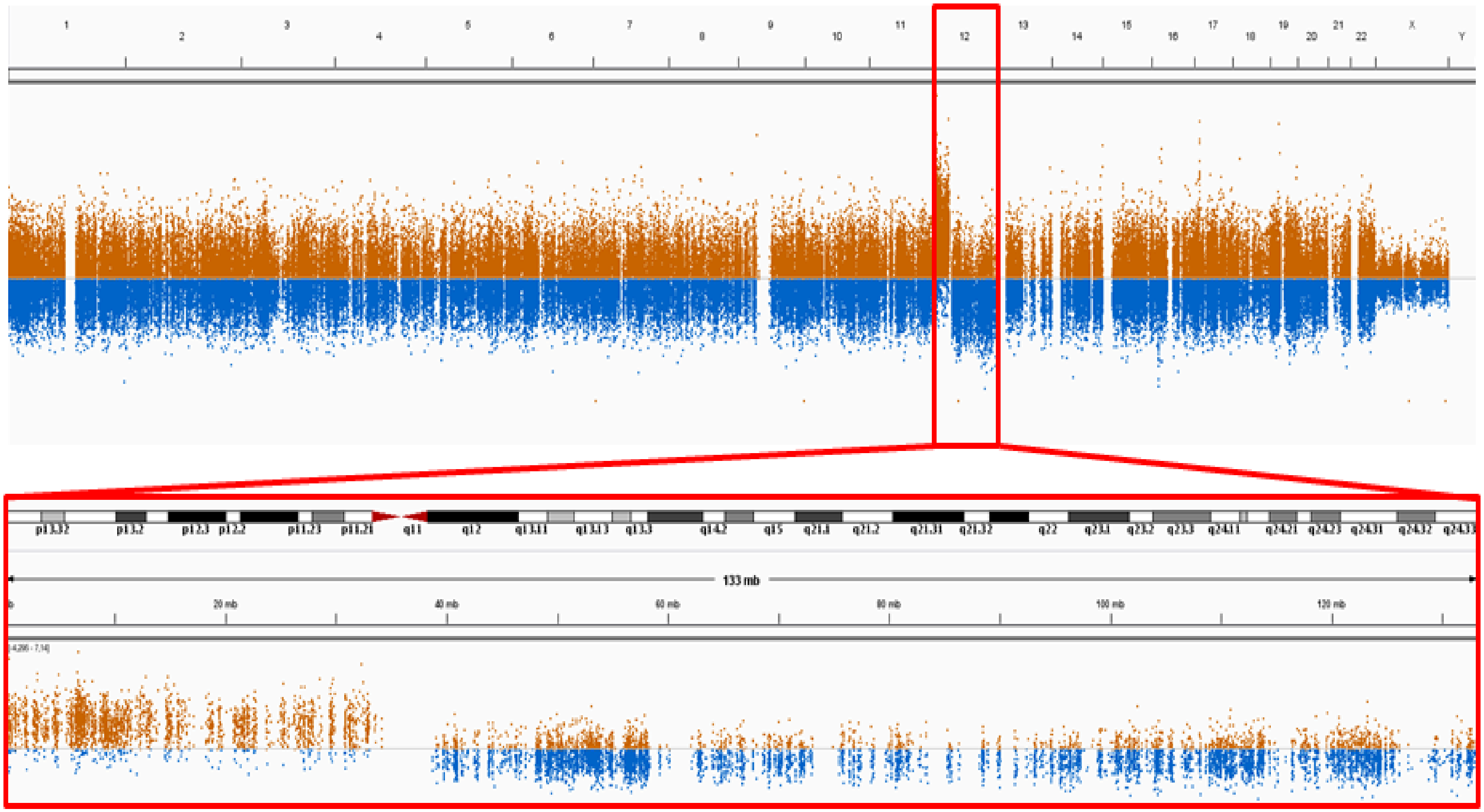

Supp. Figure S5. Normalized coverage of exon targets visualized in IGV. Upper panel shows a gain of the whole short arm of chromosome 12 (indicated by the red box), causative of Pallister Killian syndrome. Lower panel shows a zoom-in of this region. This event was detected by visual inspection of the CoNIFER bedgraph file in IGV. This CNV was called as several small gains of which only a few were located within the requested gene panel.

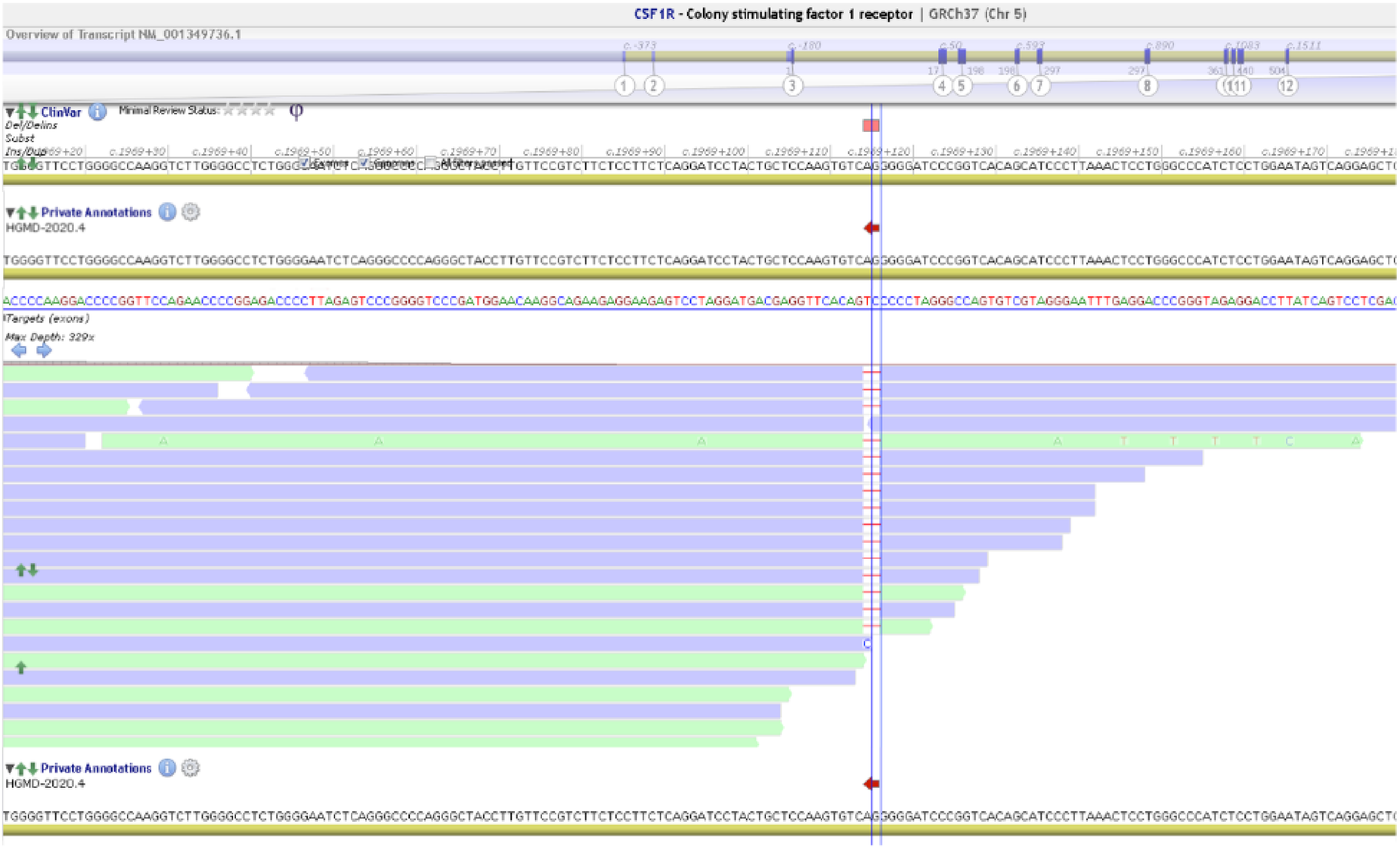

Supp. Figure S6. The pathogenic deep intronic c.1969+115_1969+116del variant in the *CSF1R* gene was discovered after a comprehensive reanalysis by the Solve-RD consortium. This variant was missed with routine WES analysis because that was restricted to the coding regions and +/- 20bp splice site regions.

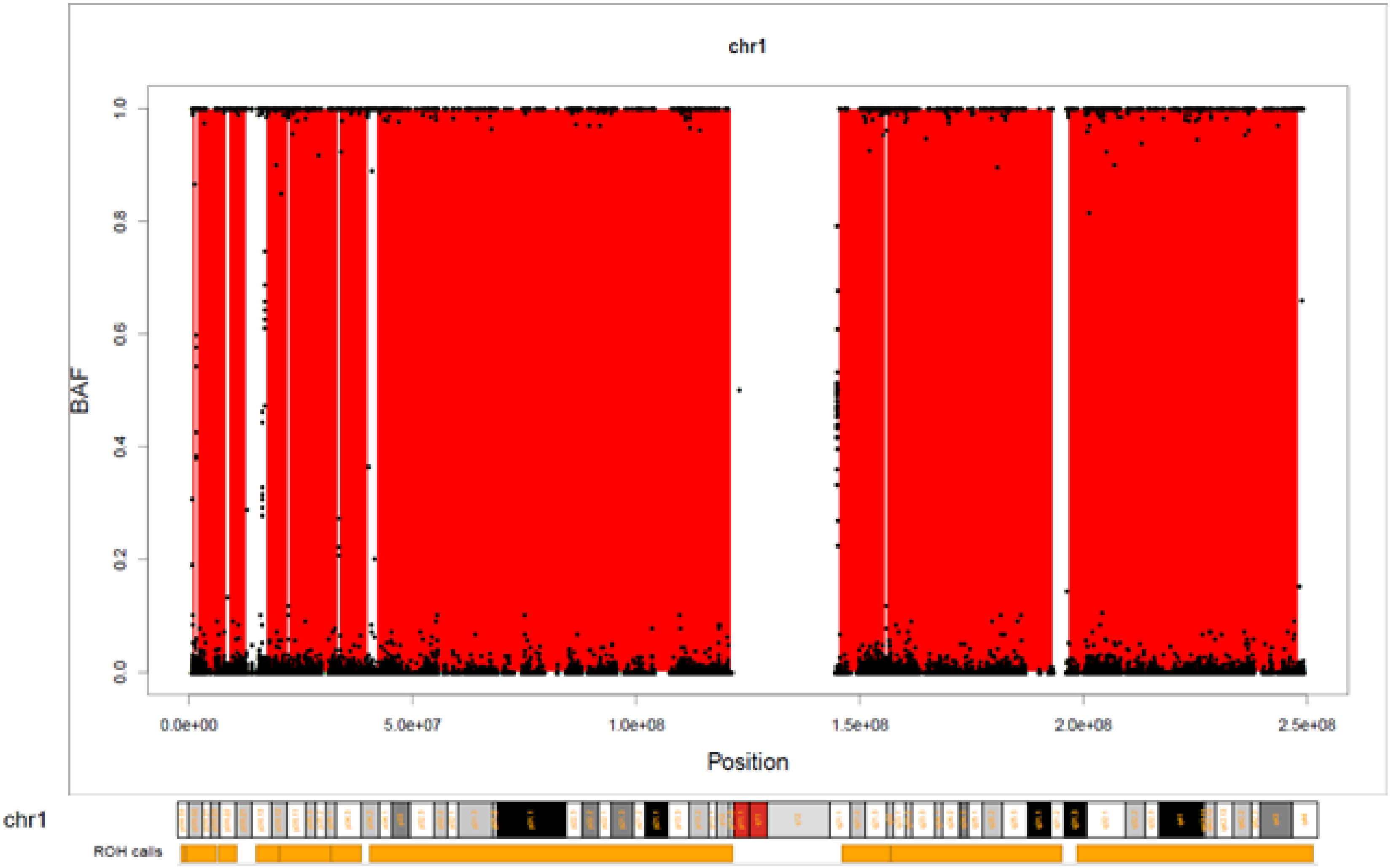

Supp. Figure S7. H3M2 ROH profile of chromosome 1 where the red regions represent the ROH calls. Profile can be interpreted as a complete homozygous chromosome 1, indicative for a uniparental isodisomy of this chromosome.

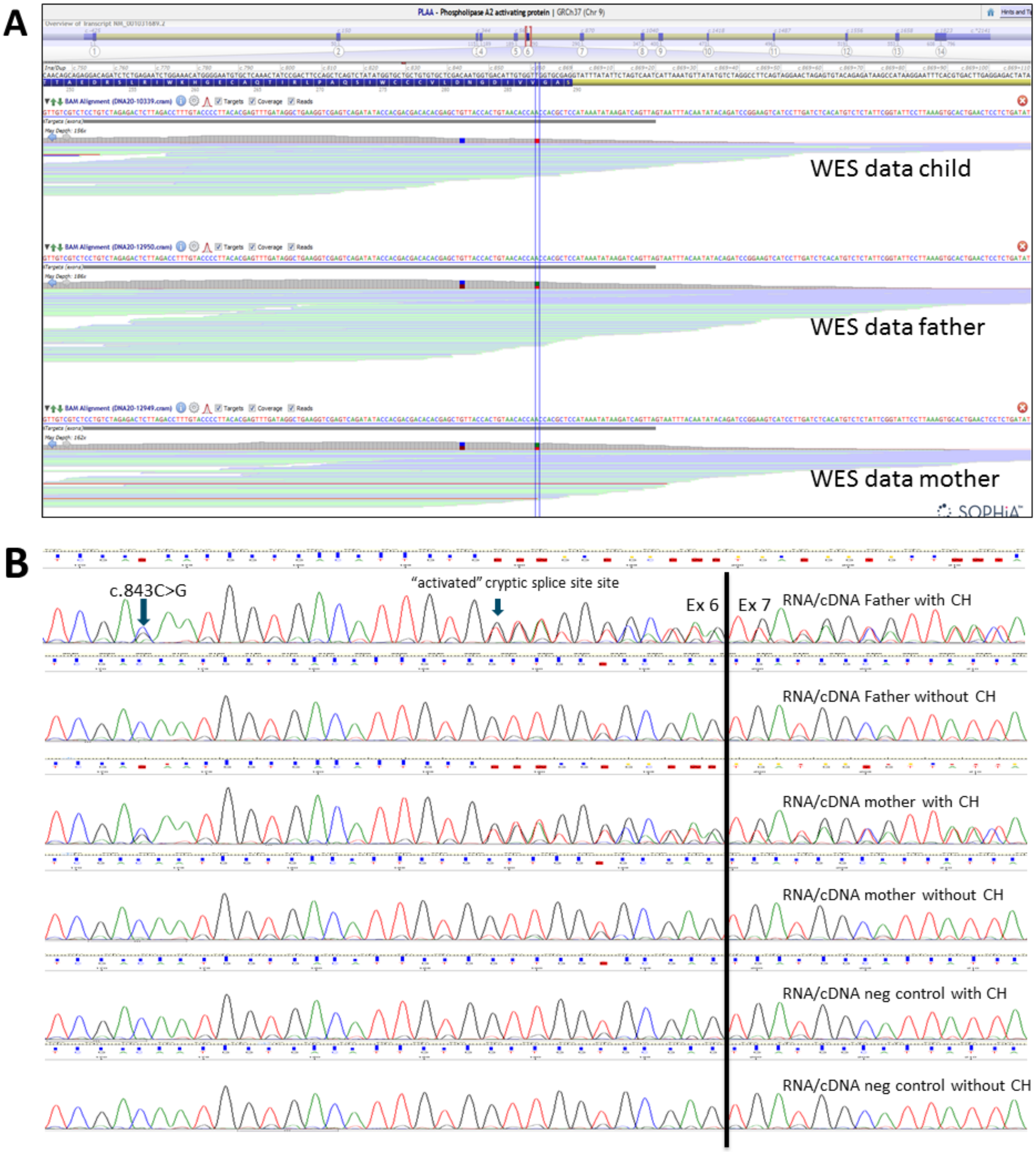

Supp. Figure S8. A) Trio exome data of exon 6 of the *PLAA* gene where two homozygous variants (Chr9(GRCh37):g.26925831A>T; NM_001031689.2:c.861T>A; p.(Val287=) and Chr9(GRCh37):g.26925849G>C; NM_001031689.2:c.843C>G; p.(Asp281Glu)) are visible in the child for which both parents are heterozygous carriers. The "silent" variant (p.(Val287=)) is predicted to affect splicing. **B)** Sequencing analysis of cDNA derived from RNA that was isolated from lymphoblastic cultures from both parents (treated with/without cycloheximide (CH)) shows that a cryptic splice donor site within exon 7 is activated by this variant, which leads to a transcript with an eleven basepair (out-of-frame) deletion that is subject to nonsense mediated decay.

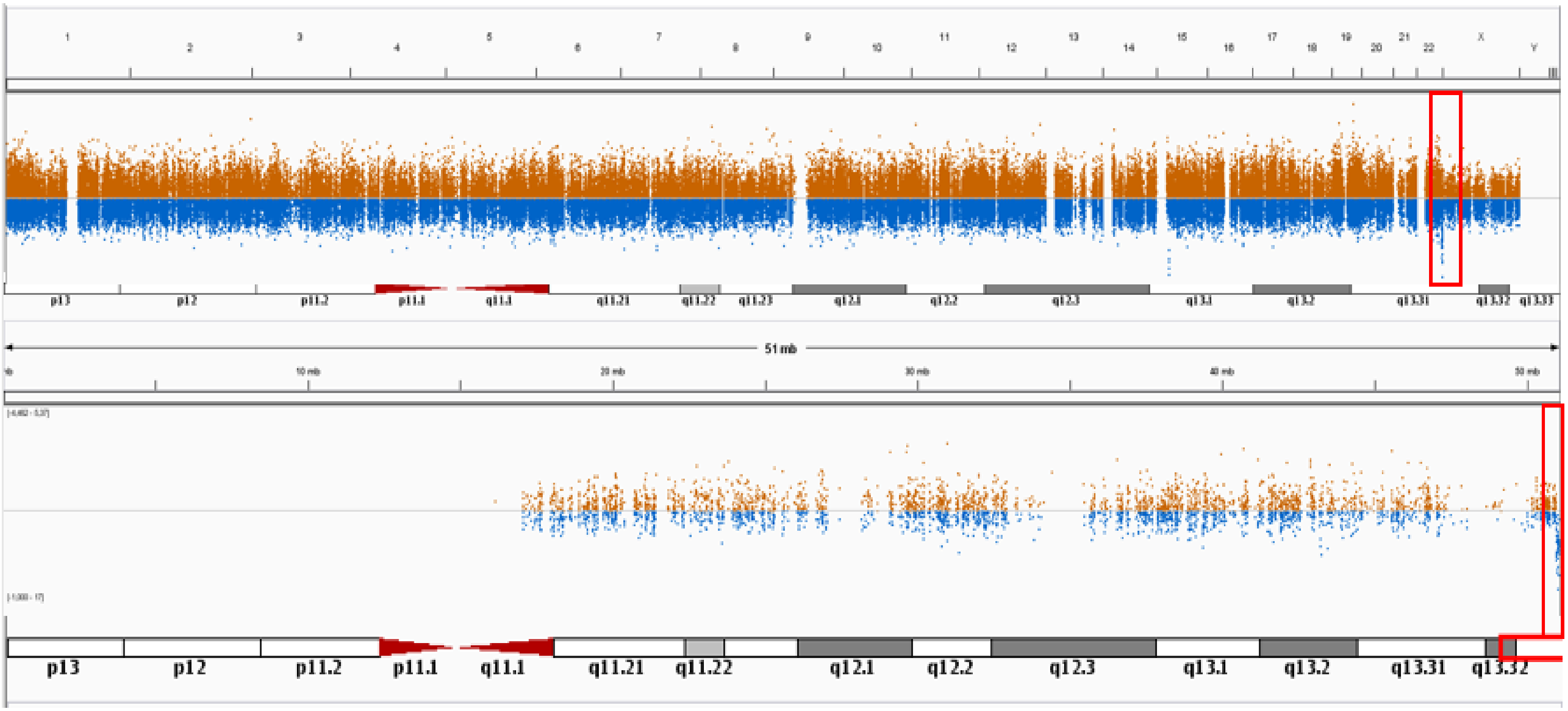

Supp. Figure S9. A ~265 kb terminal deletion on chromosome 22q13.3 can be indicative of a ring chromosome.

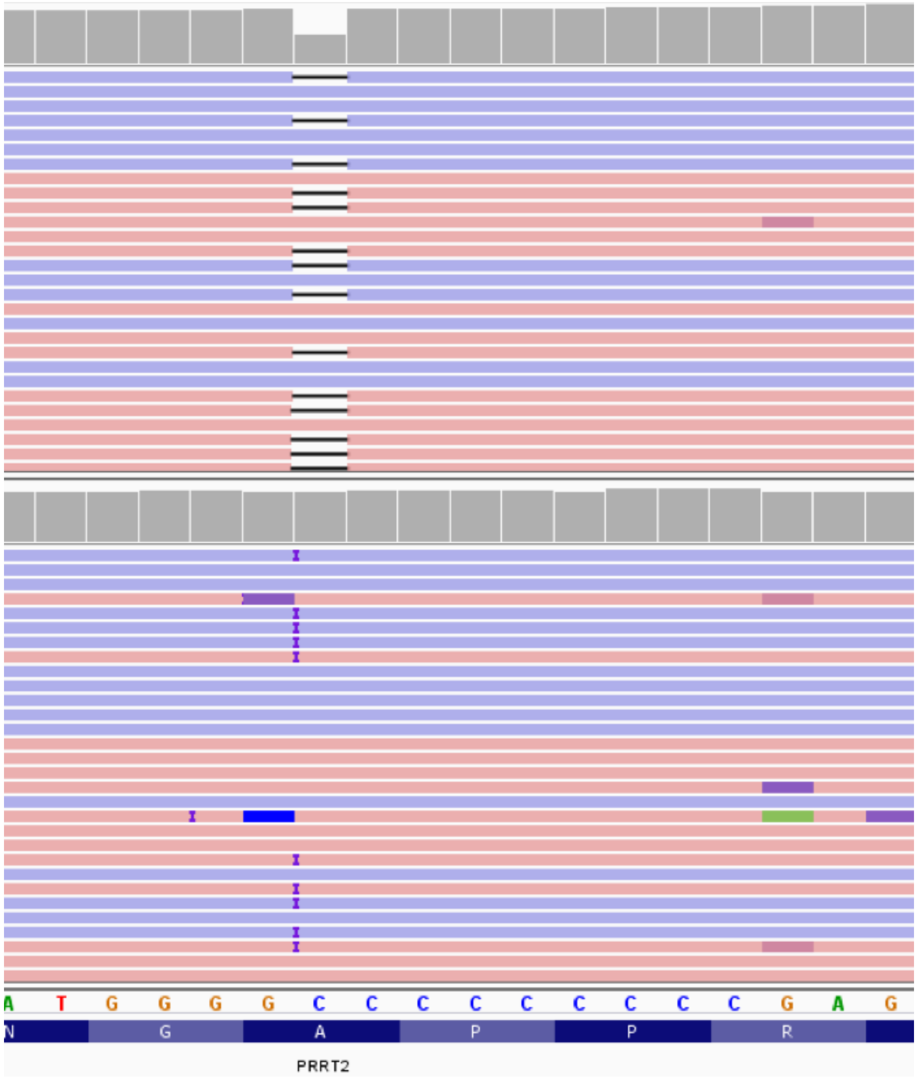

Supp. Figure S10. CRAM file alignments of two DNA samples from patients with epilepsy or episodic kinesigenic dyskinesia. One sample shows a deletion (top) and the other a duplication (bottom) of one cytosine from a stretch of nine (NM_145239.3:c.641_649) in the *PRRT2* gene (partial nucleotide and protein reference sequences shown below the CRAM files).

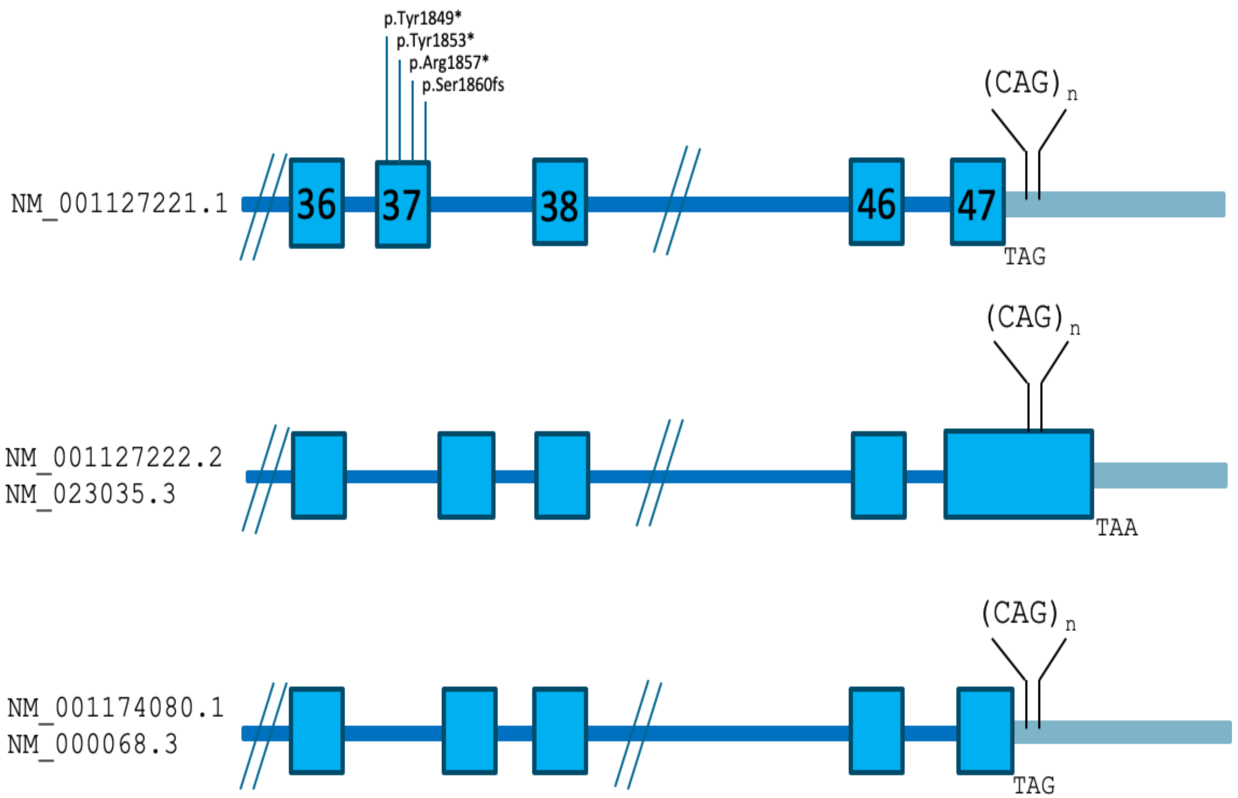

Supp. Figure S11. Schematics of the 3'end of the *CACNA1A* gene. Five different isoforms of the CACNA1A gene (*reference sequences indicated on the left*) differ in their exon content or exon length. Depicted are the exons 36-38, 46 and 47. NM_001127221.1 (*top schematic*) has an alternative exon 37 that contains loss-of-function variants (*indicated*) causative of episodic ataxia. NM_001127222.2 and NM_023035.3 (*middle schematic)* have an extended coding sequence in the last exon due to the use of another splice acceptor site in intron 46. This extended coding sequence contains the $(CAG)_n$ repeat (*indicated*), encoding the poly-glutamine tract that is expanded in spinocerebellar ataxia type 6 in these isoforms only. The termination codons in the last exon (*TAA* and *TAG*) and the untranslated region of the last exon (3'UTR) are indicated. The other differences between the isoforms reside in the part of the gene that is not shown. The schematics are not to scale.

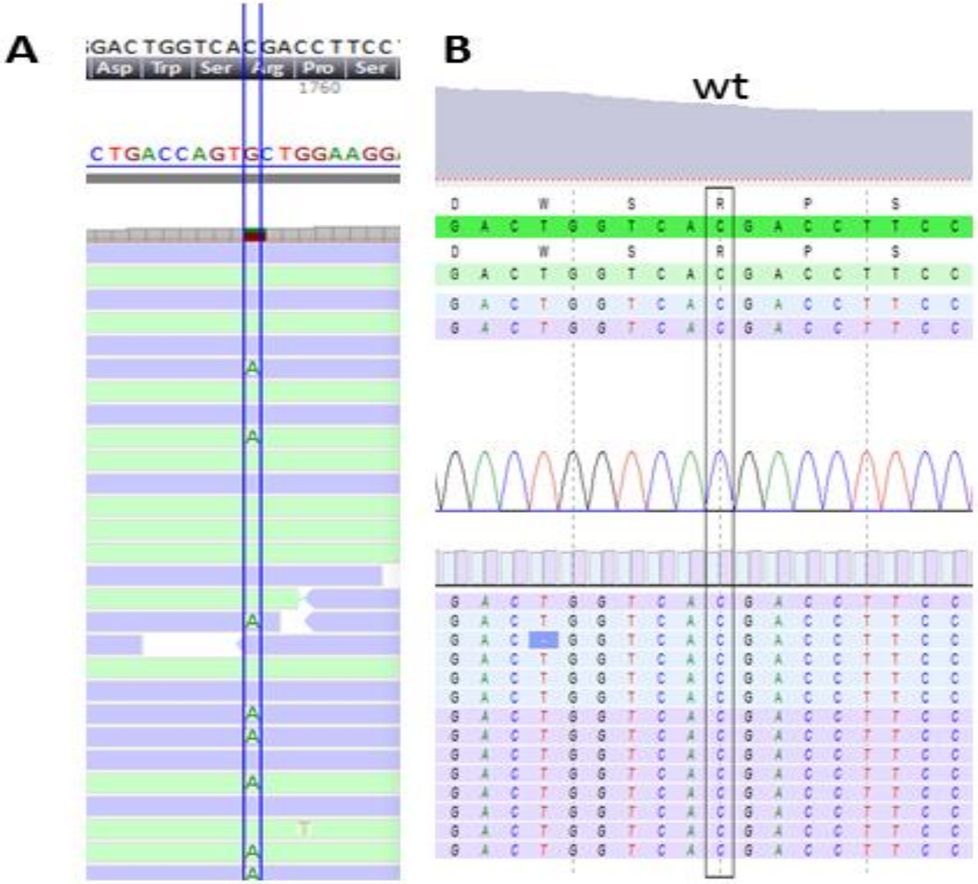

Supp. Figure S12. A) a nonsense variant in the *STRC* gene (p.(Arg1759*)) was called in approximately 30% of the reads from exome sequencing data. Since the *STRC* gene lies in a region that has a tandem duplication on chromosome 15, there is also an *STRC* pseudogene. B) The nonsense variant could not be detected when using a test that targets the *STRC* gene specifically, suggesting that the nonsense variant resides in the pseudogene.

Supplementary tables

Supp. Table S1. Summary table of lessons that we learned with relevant resources to prevent mistakes. Data Analysis sections are defined as "DA" and Variant Interpretation sections as "VI".

Discussed in section	Lessons learned
**DA-1**. Sequence quality	Perform stringent sample quality control and trend analysis.<br><br>**Resources**:<br>Qualimap (http://qualimap.conesalab.org)<br>Samtools (http://samtools.sourceforge.net)
**DA-2**. Sequence alignment: alternate contigs	Exclude alternative-contigs or use alignment algorithms that can handle alternate contigs.<br><br>**Resources**:<br>https://pubmed.ncbi.nlm.nih.gov/32232836/<br>Burrows-Wheeler Aligner (https://github.com/lh3/bwa)
**DA-3**. Variant calling: capture target file	Extend exome capture targets with at least 100 bp in order not to miss variants in coding regions.
**DA-4**. Exome CNV calling: reference pools	Create large, homogeneous sex-specific reference pools and monitor the quality of CNV calling.
**DA-5**. Annotation: Gene definitions	Update analyses and annotation datasets and re-analyze periodically.<br><br>**Resources:**<br>GENCODE (https://www.gencodegenes.org/)
**VI-1**. Visually inspect the data	Inspect variants of interest in the aligned sequencing data (BAM file)<br><br>**Resources**:<br>IGV: https://software.broadinstitute.org/software/igv/<br>Alamut Visual version 2.13 (SOPHiA GENETICS, Lausanne, Switzerland)

**VI-2**. Variants other than non-synonymous single nucleotide variants are easily missed.	Analyze exomes for other types of variants than just SNVs.
**VI-3**. Compound heterozygous variants are easily missed when one of the two is 'hiding'.	Look for a second variant when there is one variant in an AR disease gene that fits the phenotype.
**VI-4**. Remember mosaicism.	Be aware of the possibility of mosaicism and do not immediately discard variants with low VAF
**VI-5**. Think chromosomes.	Be aware of the limitations of detecting chromosomal aberration with WES and involve cytogenetic expertise when needed.
**VI-6**. Genuine disease-causing mutations may still be prevalent in population databases.	Check age the distribution of potential pathogenic variants that occur in healthy controls in Gnomad.<br><br>**Resources**:<br>https://gnomad.broadinstitute.org/<br>https://bravo.sph.umich.edu/freeze8/hg38/
**VI-7**. Distinctive clinical features may drive a correct diagnosis.	Use the phenotype to look more in depth at a (specific number of) gene(s).<br><br>**Resources**:<br>https://omim.org/<br>https://www.findzebra.com/
**VI-8**. Phenotypic information may be misleading.	Consider the possibility of phenotypic variability or incomplete phenotype information.<br><br>**Resources:**<br>https://omim.org/
**VI-9**. Non-Mendelian inheritance.	Take into account X-linked inheritance, non-penetrance and imprinting.<br><br>**Resources:**<br>https://www.geneimprint.com/site/home
**VI-10.** Be aware of isoforms, pseudogenes and gene copies.	Validate the presence and zygosity of variants in genes of which known pseudogenes exist.

Supp. Table S2. Overview of the contents of different Gencode releases according to the GENCODE website. From version V35 (August 2020) to V38 (May 2021) about 2,000 additional protein-coding transcripts were added.

**Gencode version**	**V38**	**V37**	**V36**	**V35**
Date release	5.2021	2.2021	10.2020	8.2020
# protein-coding genes	19,955	19,951	19,962	19,954
# protein-coding transcripts	86,757	86,054	85,269	84,485
